# Development of persistent gastrointestinal *S*. *aureus* carriage in mice

**DOI:** 10.1038/s41598-017-12576-0

**Published:** 2017-09-29

**Authors:** Amy Flaxman, Pauline M. van Diemen, Yuko Yamaguchi, Elizabeth Allen, Claudia Lindemann, Christine S. Rollier, Anita Milicic, David H. Wyllie

**Affiliations:** 1 Jenner Institute, University of Oxford, The Henry Wellcome Building for Molecular Physiology, Oxford, UK; 2 Oxford Vaccine Group, Department of Paediatrics, University of Oxford, CCVTM, Oxford, UK; 3 The NIHR Oxford Biomedical Research Centre, Oxford University Hospitals, Oxford, UK; 4 Jenner Institute, University of Oxford, Oxford, UK

## Abstract

One fifth to one quarter of the human population is asymptomatically, naturally and persistently colonised by *Staphylococcus aureus*. Observational human studies indicate that although the whole population is intermittently exposed, some individuals lose *S*. *aureus* rapidly. Others become persistent carriers, as assessed by nasal cultures, with many individuals colonised for decades. Current animal models of *S*. *aureus* colonisation are expensive and normally require antibiotics. Importantly, these animal models have not yet contributed to our poor understanding of the dichotomy in human colonisation status. Here, we identify a single strain of *S*. *aureus* found to be persistently colonising the gastrointestinal tract of BALB/c mice. Phylogenetic analyses suggest it diverged from a human ST15 lineage in the recent past. We show that murine carriage of this organism occurs in the bowel and nares, is acquired early in life, and can persist for months. Importantly, we observe the development of persistent and non-persistent gastrointestinal carriage states in genetically identical mice. We developed a needle- and antibiotic-free model in which we readily induced *S*. *aureus* colonisation of the gastrointestinal tract experimentally by environmental exposure. Using our experimental model, impact of adaptive immunity on *S*. *aureus* colonisation could be assessed. Vaccine efficacy to eliminate colonisation could also be investigated using this model.

## Introduction

The gram positive bacterium *Staphylococcus aureus* is an opportunistic pathogen which can cause a spectrum of diseases, ranging from relatively minor skin and soft tissue infections to serious fatal infections such as toxic shock syndrome, pneumonia, bacteraemia and sepsis. Increased effort in the field of *Staphylococcus aureus* research over the last decade has not yet resulted in a reduction in morbidity due to *S*. *aureus* associated disease^1^. Rather, the health and economic burden associated with *S*. *aureus* infection and disease is increasing^2^, largely due to the rise in antibiotic resistant strains, which pose a threat in both the community and hospital settings^3^.

*S*. *aureus* is a common component of normal human flora, with approximately 20–25% of the adult population described as persistent nasal carriers^4^, and a further 10–25% classified as intermittent carriers^5,6^. Although frequently considered a skin coloniser, *S*. *aureus* is a common member of the gastrointestinal (GI) tract flora: detectable GI tract carriage is about 60% in nasal carriers^7,8^. However, this may be an underestimate, as *S*. *aureus* is a minority population in the bowel and its detection may be influenced by culture techniques used^6^. A recent study has highlighted the possible underestimation of *S*. *aureus* bowel carriage as a reservoir for subsequent infection^9^. Culture of other sites (groin, axilla and throat) in addition to the nares also adds to the carriage yield of *S*. *aureus* ^6^. Human decolonisation/recolonization studies indicate that ‘colonisability’, at least in adults, is a persistent state with a propensity towards recolonization with the same, rather than unrelated, *S*. *aureus* strains^10^.

Multiple prospective cohort studies have shown that carriers are at about three fold higher risk of invasive *S*. *aureus* disease than non-carriers, most commonly due to their carried strain^1,4^. This association is causal, since transient decreases in the concentrations of carried *S*. *aureus* achieved using antimicrobials reduce *S*. *aureus* disease risk^11^. Development of interventions which reduce carriage would therefore be clinically useful and highly desirable for reducing the risk of invasive disease which can lead to hospitalisation and even death^12^. Although intervention studies in humans have been conducted^13^, larger scale experimentation would be aided significantly by a relevant animal model.

*S*. *aureus* prevalence is documented in farm animals where it can cause sub-clinical mastitis in cows, goats and sheep^14^. These populations also represent zoonotic sources of colonisation of human populations^15^. Non-human primates have been shown to be oro-pharyngeal (lemurs and captive chimpanzees) and faecal carriers (wild chimpanzees)^16^. Both farmed and companion rabbits can be colonised by a strain similar to a common human clone^17,18^ and host adaptation from human to rabbit was enabled by a single nucleotide mutation^17^. Piglets can be colonised experimentally^19^. Faecal colonisation has also been described in small wild mammals^20^. Thus, *S*. *aureus* strains can colonise multiple mammalian species. Recent studies have investigated the impact of the microbiota in *S*. *aureus* carriage in both pigs^21^ and mice^22^.

Although short-term (up to 28 days) experimental intranasal colonisation in rodents has been established^23,24^, until recently mice were not thought to be long-term (>28 days) or natural hosts of *S*. *aureus*. However, an outbreak of preputial gland infections in an animal facility in New Zealand in 2008 led to the discovery that the affected animals were persistently colonised with a mouse-adapted *S*. *aureus* strain^25^. The same authors have recently published data from a survey of commercial mouse breeding organisations indicating that *S*. *aureus* from nine clonal complexes can persist in mouse colonies^26^. To date, experimental long-term persistent carriage models in cotton rats^24^, but not mice have been reported^27^.

Here we describe a natural model of *S*. *aureus* GI tract colonisation in mice and characterise the strain responsible. Further, we demonstrate a novel, needle- and antibiotic-free methodology to induce experimental *S*. *aureus* gastrointestinal colonisation. We show that a proportion of genetically identical mice become persistent carriers, maintaining colonisation for 28 days and longer, while a proportion lose carriage, akin to the situation in humans. We propose the model demonstrated will have application in the development of *S*. *aureus* vaccines and in studies investigating the basis of *S*. *aureus* carriage.

## Results

### Mice are naturally colonised with *S*. *aureus*

Female BALB/c mice obtained from Harlan (Blackthorn, UK) were found to be faecal carriers of *S*. *aureus* upon arrival at our animal facility in December 2013. We subsequently implemented routine stool sampling of BALB/c mice upon arrival. From December 2013 to May 2015, 416/433 (93.91%) of the 6–8 week old female BALB/c mice were found to be faecal carriers of S. *aureus* when sampled within 7 days of arrival.

**Figure 1:**
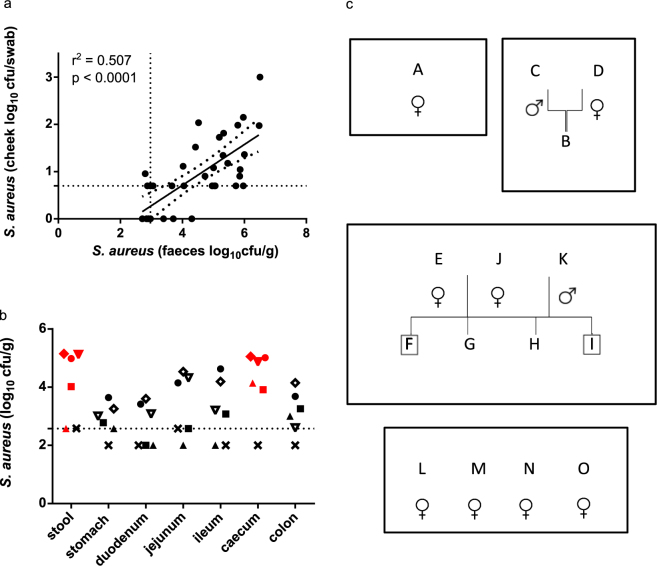
*S*. *aureus* naturally colonises the GI tract of mice. (**a**) *S*. *aureus* bacterial recovery from stools and cheek swabs in 36 animals. (**b**) *S*. *aureus* bacterial recovery from GI tract sections in 6 animals. (**a**,**b**) *S*. *aureus* bacterial recovery from stool samples, cheek swabs and sections of GI tract from female BALB/c mice from Harlan was determined by plating onto selective agar. Horizontal dotted lines in (**a**) and (**b**) represent the limit of detection of the culture assay. For cheek swab samples, negative samples are plotted as if 1 colony/g colony was detected. For faecal and organ samples, negative samples are plotted as if 100 colonies/g were detected. Samples coloured red were s*pa* typed and found to be t084. (**c**) *S*. *aureus* is efficiently transmitted from parents to offspring. Stool samples were obtained from an in-house breeding colony of BALB/c mice. Each box represents a cage within the colony. A, C, D, E, J & K are breeders, B, F, G, H, I are pre-weaned pups and L, M, N, O are weaned pups (samples from parents of these animals were not available). *S*. *aureus* was recovered from stools of all animals except F and I (indicated by boxed letters).

Nasal swabbing of humans is routinely used in studies assessing *S*. *aureus* carriage status^4,28^. As murine faecal sampling and cheek swabbing are much easier technically than murine nasal swabbing, all three methods were compared in a preliminary experiment. *S*. *aureus* bacterial recovery (cfu) from cheek swabs and faecal samples showed strong positive correlation (*p* < 0.0001, r^2^ = 0.507) (Fig. 1a). However, faecal sampling appeared more sensitive than either nasal or cheek culture (Supplementary Table 1), and thus was selected as the measure of murine *S*. *aureus* carriage in the rest of the study.

*S*. *aureus* was recovered from GI tract sections in faecal carriers (Fig. 1b) and *S*. *aureus* bacterial recovery in different parts of the GI tract positively correlated with faecal bacterial recovery (Supplementary Fig. 1). Since the majority of female BALB/c mice obtained from the supplier were *S*. *aureus* carriers, we investigated whether *S*. *aureus* colonisation existed in a separate, in-house breeding colony of BALB/c mice (Fig. 1c). We detected faecal carriage of *S*. *aureus* in 3/5 pre-weaned pups and 4/4 post weaning pups and 6/6 adults. This indicates that acquisition of carriage occurs early in life, and that *S*. *aureus* is efficiently acquired by offspring within a breeding colony.

### The colonising *S*. *aureus* strain is clonal and is related to human ST15

*spa* typing was performed on *S*. *aureus* isolates from stools of 4 pups from the in-house breeding colony and were s*pa* type t2177. *spa* typing was also performed on *S*. *aureus* isolates from stools of mice obtained from Harlan. In total 91 isolates from 62 different BALB/c mice obtained were *spa* typed and found to be t084. These samples were from multiple different deliveries from Harlan and include isolates from stool and caecum (Fig. 1b). Two further isolates from stools from two different mice were found to be spa types t120 and t346 respectively, which each differ from t084 by only one repeat. These findings indicated that different *S*. *aureus* sequence types are capable of colonising mice, as t2177 and t084 are not related, although one clone may dominate particular facilities.

*S*. *aureus* isolates from four mice housed in the same cage were further analysed. All four were *spa* type t084 and showed the same antibiotic resistance pattern including penicillin resistance, methicillin sensitivity and sensitivity to other commonly used antibiotics. Illumina-based whole genomic sequencing of these isolates indicated that all four were identical except for one isolate which differed from the other three by 17 single nucleotide polymorphisms, scattered across the core genome. Therefore, a clade of closely related *S*. *aureus*, which we termed ‘SaF’, was colonising the GI tract of BALB/c mice arriving at our animal unit from Harlan. One of the three identical isolates was selected to be used in all further experimental colonisation work and named ‘SaF_1’. *In silico* multilocus sequence typing showed that the 4 SaF clade isolates belong to sequence type (ST) 15.

**Figure 2:**
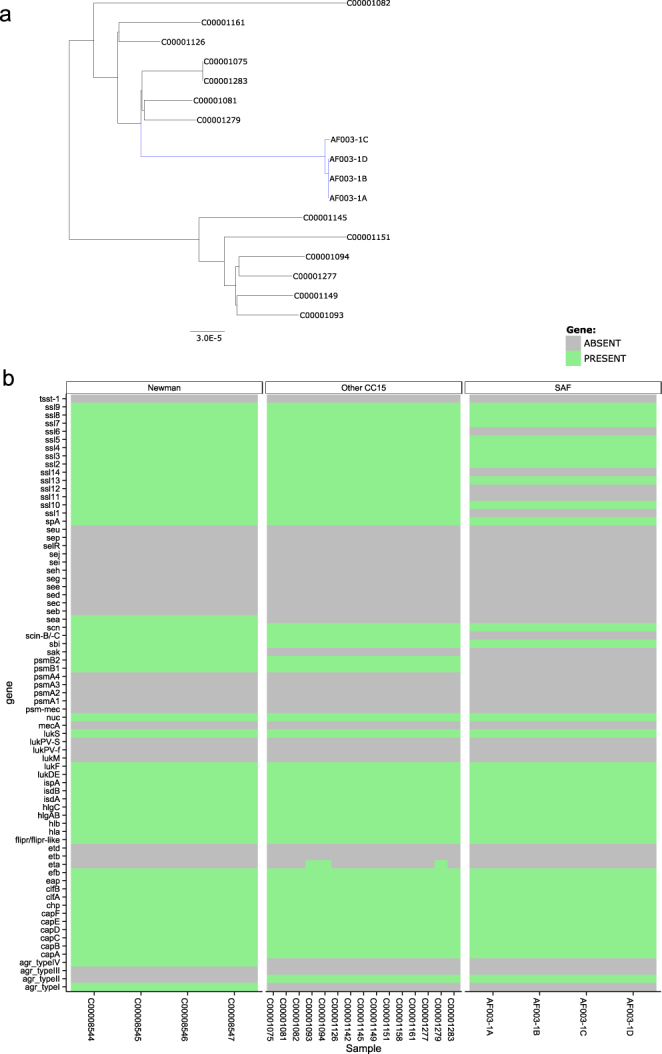
Characteristics of SaF strain revealed from whole genome sequencing. WGS was performed on *S*. *aureus* isolated from stools from 4 female BALB/c mice, which we termed SaF. Isolates were labelled AF003-1A, -1B, -1C and 1D. (**a**) Maximum likelihood tree showing relationship to human ST15 isolates (SaF clade highlighted in blue). Scale bar refers to per base substitution rate. (**b**) Gene presence/absence table comparing ‘SaF’ isolates to human CC15 isolates and multiple independent sequencing of the standard laboratory strain Newman. Grey indicates absence and green presence of the gene.

To assess its possible origin, we compared the SaF clade isolates with a set of fifteen ST15 human isolates, obtained from nasal screening at GP practices in the UK, previously described by Everitt *et al*.^29^. A maximum likelihood phylogenetic tree was constructed, and evidence of recombination between the human and mouse strains sought using ClonalFrameML. The maximum likelihood tree is shown (Fig. 2a). There was no evidence of recombination (not shown). We therefore used BEAST to reconstruct the likely evolutionary relationship between our four isolates. In the absence of any other published estimates of an ST15 clock rate, and insufficient clock signal in the samples available to us to estimate this directly, we used a published estimate of the *S*. *aureus* molecular clock^30^. This indicated that among our four SaF isolates, the one differing strain diverged from the others approximately 1.6 years previously (95% credible interval 1.0–2.4 years). Assuming a single species jump event, this approach also indicated that the isolated murine strains may have diverged from the related human strain about 24.2 years (95% credible interval 22.2–26.2 years) prior to the isolation date.

The 4 SaF clade isolates were mapped to reference genome MRSA252 and compared with the documented variation in human ST15 isolates from a published collection^29,31^, representative of human *S*. *aureus* diversity. This indicates that the SaF clade is similar to, and maps within, identified human ST15 isolates (Fig. 2a). To assess diversity in the accessory genome, gene presence/absence estimation was performed against a 73 gene panel *in silico* (Fig. 2b) which demonstrated that the SaF clade isolates are similar to human ST15. Like human ST15 isolates, the SaF clade lacks some superantigen genes^32,33^, while others are present in the ST15 human strains but absent in SaF, suggestive of gene loss. Similarly, genes associated with the immune evasion cluster (IEC, e.g. *scn*) were missing. We concluded that SaF was similar to human *S*. *aureus* ST15 clones, but lacked some genes associated with mobile elements in human derived clones. Overall, we considered the genomic analysis compatible with the strain to be of recent human origin, potentially acquired from animal care takers in the breeding facility/supplier.

### *S*. *aureus* gastrointestinal carriage can persist over months

**Figure 3:**
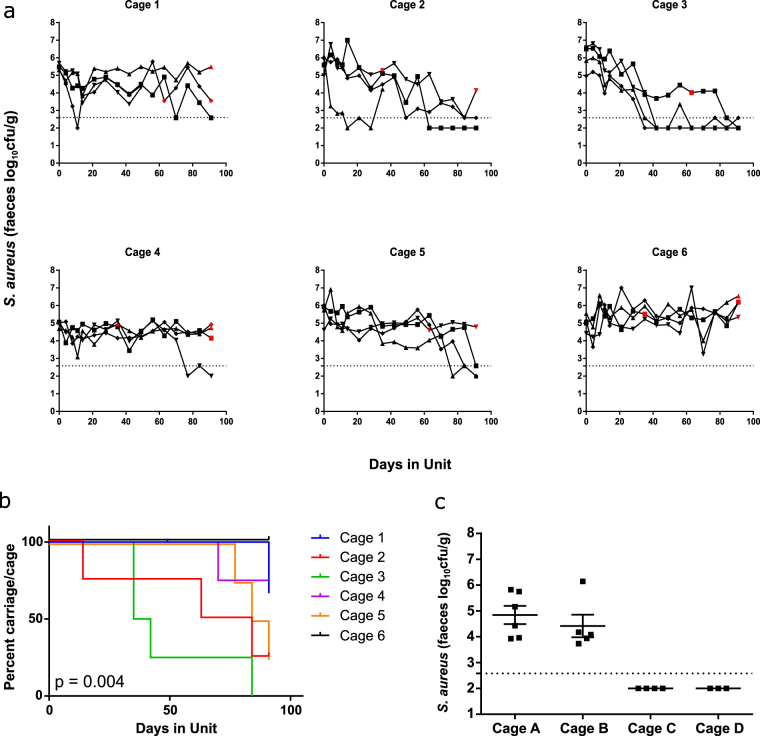
Longitudinal monitoring of SaF carriage. (**a** and **b**) *S*. *aureus* carriage levels were monitored by stool sampling in 24 female BALB/c mice, randomly allocated 4 per cage, for 3 months. (**a**) Carriage levels are shown per mouse within a cage. Samples coloured red were s*pa* typed and found to be t084. (**b**) Kaplan-Meier analysis for loss of carriage per cage: *p* value given for log rank (Mantel-Cox) test. Adverse events occurred as follows – one mouse in Cage 1 had to be culled at 49 days and one mouse in Cage 2 had to be culled at 35 days due to unrelated illness. (**c**) Cross-section of *S*. *aureus* carriage in older mice. *S aureus* carriage levels were tested by stool sampling in 18 female BALB/c mice from Harlan 13 months after arrival at the animal unit. Carriage levels are shown for individual mice at one time point only. Horizontal dotted lines in (**a**) and (**c**) represent the limit of detection of the culture assay. Negative samples are plotted as if 100 colonies/g were detected.

We investigated dynamics of *S*. *aureus* carriage in mice colonised prior to arrival at our animal facility (naturally colonised mice). Bacterial recovery from mouse stools was monitored longitudinally. Groups of four to six mice were randomly assigned to individually ventilated cages and *S*. *aureus* carriage was monitored for at least 90 days in three independent experiments. Data from all experiments are presented: Fig. 3 shows Experiment 1 (24 mice) and Supplementary Fig. 2 shows experiments 2 and 3 (12 mice in each).

Different patterns of *S*. *aureuS*. *aureus* carriage were observed across different cages (Fig. 3). For example, animals in Cage 3 had all lost *S*. *aureus* carriage by the end of the experiment (Fig. 3a), whereas those in Cage 6 maintained carriage levels seen at arrival throughout the experiment. Loss of carriage was defined as the time point at which S. *aureus* carriage was lost or detectable by enrichment only at that, and the following, time point. Time to loss of *S*. *aureus* carriage was assessed using survival analysis. Time to loss of carriage was significantly different across the 6 cages of the experiment shown in Fig. 3b (p = 0.004, assessed using a Log rank (Mantel-Cox) test). 6 mid-experiment isolates and 11 isolates available at the end of the experiment (from mice which had not lost carriage) were *spa* type t084 (Fig. 3a). Differences in *S*. *aureus* carriage between cages and maintenance of *spa* type t084 were confirmed in two other experiments (Supplementary Fig. 2). These data imply that each cage is a microenvironment within which the dynamics of *S*. *aureus* carriage differs but that the same colonising strain is maintained throughout experiments.

As the mice used in the experiments described above were aged 6 weeks to 4–5 months, we investigated whether *S*. *aureus* carriage existed in an apparently dichotomous state in older mice. We performed a cross-sectional analysis of *S*. *aureus* carriage in 18 BALB/c mice 13 months after arrival in the animal unit, and found that *S*. *aureus* carriage was present in 2 out of the 4 cages tested (Fig. 3c). This finding is compatible with the polarisation we observed in younger animals persisting for up to 12 months and possibly longer.

### Co-housed mice can transmit *S*. *aureus*

**Figure 4:**
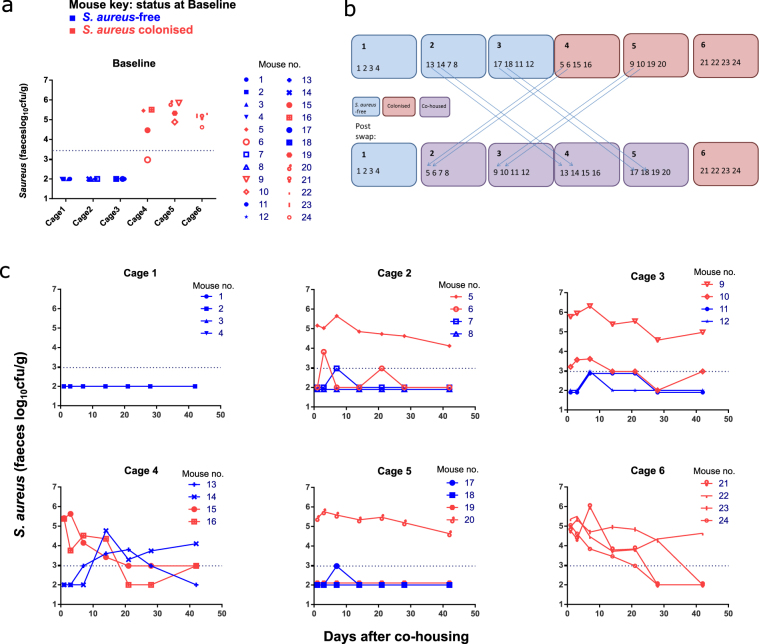
Co-housing to investigate transmission between *S*. *aureus-*free and colonised mice. 24 female BALB/c mice were monitored for GI tract colonisation by stool sampling longitudinally. Mice were housed in cages of 4 at all times. At first *S*. *aureus*-free mice were housed in cages 1 to 3 and *S*. *aureus* colonised mice in cages 4 to 6. After taking baseline stool samples, a ‘cage-swap’ was performed: 2 *S*. *aureus*-free mice were co-housed with 2 colonised mice in Cages 2–5. No swapping occurred in Cages 1 & 6; these acted as negative and positive controls, respectively. (**a**) Baseline carriage levels for individual mice. (**b**) Experimental design. (**c**) Carriage levels after ‘cage-swap’. Individual mice labelled 1–24 throughout experiment. Horizontal dotted lines in (**a**) and (**c**) represent the limit of detection of the culture assay. Negative samples are plotted as if 100 colonies/g were detected.

To investigate whether naturally colonised adult mice can transmit *S*. *aureus* to other mice, we co-housed *S*. *aureus-*free and colonised BALB/c mice (both from Harlan). Initially, we housed 4 mice in each cage, all of which were either *S*. *aureus*-free (3 cages, 12 mice) or *S*. *aureus* colonised (3 cages, 12 mice). After taking baseline stool samples (Fig. 4a), we performed a ‘cage-swap’. This resulted in 4 cages of co-housed mice: 2 *S*. *aureus*-free animals were housed with 2 colonised animals (by swapping 2 mice from a *S*. *aureus*-free cage to a colonised cage and vice versa). One cage with only *S*. *aureus*-free mice and one cage with only colonised mice were used as controls throughout (no swapping) (Fig. 4b). We monitored *S*. *aureus* carriage levels in all mice for 45 days (Fig. 4c).

We observed *de novo* acquisition of *S*. *aureus* in some initially *S*. *aureus*-free animals (Fig. 4c, mice 13 & 14); however, other co-housed mice (7, 8, 11, 12, 17 & 18) demonstrated either low-level carriage, which was only transiently detected, or no acquisition of carriage. Thus, under the conditions found in adult cages, transmission can occur, albeit infrequently.

### Short term colonisation by oral gavage

**Figure 5:**
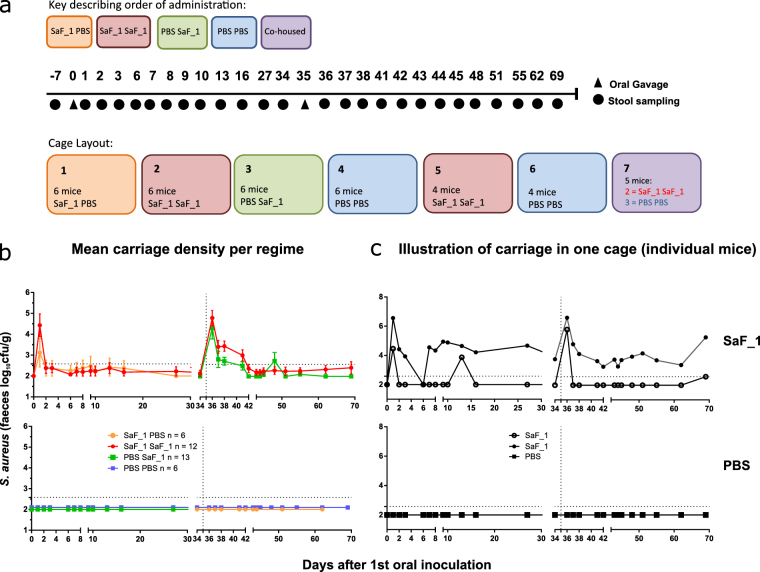
Experimental gastrointestinal colonisation of *S*. *aureus-*free mice with SaF. 37 Female BALB/c mice were confirmed to be *S*. *aureus*-free by stool culture. Animals were given 10^8^ cfu SaF_1 or PBS by oral gavage and carriage levels were monitored by stool sampling. Each mouse received two oral gavage treatments at 0 days and 35 days (indicated by a vertical dotted line) with SaF_1 or PBS. (**a**) Experimental Design and Timescale (**b** and **c**) Top panels show SaF_1, bottom panels show PBS exposure. (**b**) Mean carriage levels shown per regime. 6–13 mice per regime. (**c**) Individual carriage levels for one cage containing 5 mice: 2 received 2 doses of SaF_1, 3 received 2 doses of PBS. Horizontal dotted lines represent the limit of detection of the culture assay. Negative samples are plotted as if 100 colonies/g were detected.

In order to investigate whether the SaF_1 strain was able to colonise the GI tract of *S*. *aureus*-free mice experimentally, 37 certified *S*. *aureus*-free BALB/c mice were obtained from Taconic, and their *S*. *aureus*-free status was confirmed by stool sampling upon arrival. Faecal carriage was monitored after mice received oral gavages at Day 0 and at Day 35 (Fig. 5a) of either PBS (Fig. 5b bottom panels) or 10^8^ cfu of SaF_1 in the same volume of PBS (Fig. 5c top panels) Faecal carriage of very short duration was induced in mice receiving SaF_1, with all mice except two in the group receiving two doses of SaF_1 losing faecal carriage within 7 days of each gavage (Fig. 5). SaF_1 carriage was detectable in these two mice up to 133 days after first oral gavage, although they were housed in different cages. *S*. *aureus* isolates from their stool, nose and cheek from 84 days after first oral gavage were confirmed as *spa* type t084, and therefore presumed to be SaF_1. One of the mice persistently carrying SaF_1 was housed in a cage with other mice which received PBS twice (Fig. 5c), indicating that, at least in some animals, colonisation can persist without repeated re-exposure from others in the cage. At the end of the experiment, 203 days after the first oral gavage, we were unable to detect *S*. *aureus* colonies in segments of GI tract from 3 mice which received SaF_1 twice and subsequently lost faecal carriage. This implies that loss of faecal carriage is indicative of loss of carriage throughout the GI tract. Taken together, these results demonstrate that short term exposure to *S*. *aureus* can result in persistent colonisation, but at a low frequency. It also supports the previous experiment (Fig. 4) indicating that co-housing with a colonised mouse is not sufficient to consistently initiate long-term colonisation in adults.

### Experimental colonisation by environmental contamination

**Figure 6:**
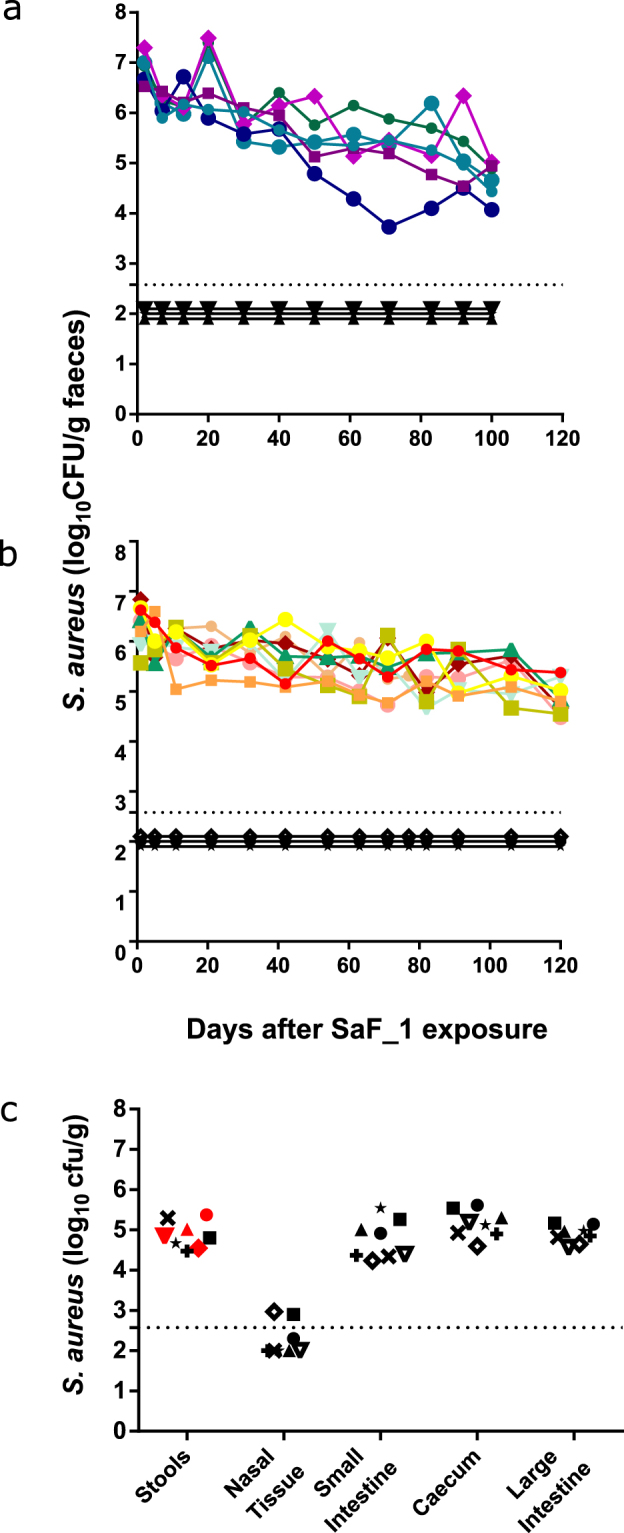
Experimental *S*. *aureus* colonisation of *S*. *aureus*-free mice by environmental contamination. Mice were confirmed *S*. *aureus*-free by stool sampling. 6 mice colonised as neonates (**a**) received 4 SaF_1 doses from -1 to 14 days old, whilst 9 animals colonised as adults (**b**) received 1 dose at 6 weeks. *S*. *aureus* colonisation levels were monitored by stool sampling for at least 100 days. Each coloured symbol represents an individual mouse which received SaF_1 treatment. Black symbols represent control animals which received PBS treatment (3 per experiment). (**c**) *S*. *aureus* recovery from 8 mice colonised with SaF_1 as adults at 100days post colonisation (various samples throughout GI tract, one mouse culled prior to experiment end point due to unrelated illness). Horizontal dotted lines represent the limit of detection of the culture assay. Negative samples are plotted as if 100 colonies/g were detected. Samples coloured red were *spa* Typed and found to be t084.

Since colonisation by oral gavage resulted in short-lived *S*. *aureus* GI tract colonisation, we speculated that intense and persistent environmental exposure might be required to initiate carriage efficiently. Therefore, a *S*. *aureus* SaF_1 culture was then sprayed into the cage environment of certified *S*. *aureus*-free BALB/c mice (confirmed by stool sampling upon arrival). A preliminary experiment involved spraying *S*. *aureus* SaF_1 culture into empty cages showed that *S*. *aureus* could be recovered from ‘contaminated’ cages up to 52 hours post spraying, but no longer (data not shown). Using the spraying methodology, both neonates (n = 6) and adult (n = 9) BALB/c mice were successfully colonised, with faecal colonisation being maintained for at least 100 days in some animals (Fig. 6a,b respectively). In parallel, certified *S*. *aureus*-free BALB/c mice housed in other cages were sprayed with PBS only to act as negative controls. These animals remained *S*. *aureus*-free throughout the experiments. *S*. *aureus* was also isolated from other GI sites and nasal tissue at 100 days post treatment (Fig. 6c). Isolates from stools of 4 mice at 100 days post treatment were *spa* type t084 (Fig. 6c) indicating that the strain administered persists throughout the experiment. Thus, we have established a novel methodology for establishing *S*. *aureus* GI tract colonisation which is needle-free and does not require antibiotics.

## Discussion

In view of the potential benefits of a reproducible, controlled small animal model of *S*. *aureus* colonisation, we investigated *S*. *aureus* colonisation and dynamics in mouse populations. We described *S*. *aureus* GI tract colonisation, characterised the strain responsible and showed that colonisation can be maintained in mouse colonies for many months. Given that mice aged 6 weeks arrived at our animal facility already colonised and that pre- and post-weaning pups and parents within a breeding colony were also colonised, we hypothesise that mother-to-pup transmission occurs. This conclusion is supported by observational studies performed by Schulz *et al*.^26^, who surveyed *S*. *aureus* strains colonising breeding facilities worldwide and showed the establishment of persistent colonisation of neonatal mice with CC1 and CC88 isolates.

Following exposure early in life, genetically identical animals dichotomised into carrier and non-carrier populations (Fig. 3, Supplementary Fig. 2); similar dichotomisation appears to occur in humans^10^. In humans, universal exposure occurs in childhood^34^. Human experimental colonisation with strain 502 A performed in the 1960s demonstrated efficient neonatal colonisation^35–37^. Subsequent molecular studies confirmed extensive mother-child strain sharing in the community^38^. Thus, colonisation in humans (as in mice) can occur early in life, with the mother being a likely source.

Studies have proposed that humans with and without persistent *S*. *aureus* carriage differ in their rates of loss following exposure and that the local human environment may be critical in maintenance of carriage^39^. Similarly, household environmental contamination has recently been associated with an increased rate of recurrent infecton in CA-MRSA^40^. Our observation that genetically identical mice can develop a persistent carrier or non-carrier state (Fig. 3) suggests that similar acquired alterations in loss rate may occur in other animals. In particular, we have shown that individual animals can persistently carry *S*. *aureus* without contact with other colonised mice, but that carriage patterns of mice within an individual cage are related to each other (Fig. 3, Supplementary Fig. 2). Thus, the environment may contribute to the carriage patterns observed in individuals.

In addition to monitoring *S*. *aureus* colonisation in mice which were naturally colonised, we used 3 approaches to attempt to colonise *S*. *aureus* naïve animals. Housing *S*. *aureus* naïve adult mice with *S*. *aureus* colonised mice only resulted in 2/12 animals becoming colonised. Likewise, colonisation attempts using oral gavage in *S*. *aureus* naïve adult mice, whilst successful in establishing very short-term (<7 days) colonisation, only resulted in long-term (>28 days) colonisation in 2/24 mice. The third approach, contamination of the environment, proved successful in establishing long-term colonisation in 6/6 pre-weaned mice and 9/9 adult mice. Therefore, we propose that different levels of exposure at different stages of development may affect whether a mouse can become colonised. The biology surrounding how persistent colonisation is established using the environmental contamination method is an area for further investigation. An additional area for investigation concerns study of whether the determinants of gastrointestinal carriage are the same as for nasal carriage. Using the methods described here, the frequency of positivity, and the concentrations of bacteria recovered, are much lower from nasal samples than from stool samples. Whether this reflects technical or biological phenomena is remains unclear.

Nevertheless, we have demonstrated that our highly efficient needle- and antibiotic-free model involving experimental exposure leads to the initiation of *S*. *aureus* GI carriage in mice. Our model presents a less invasive procedure compared to currently used nasal colonisation methods^23,24,41–43^. Our experimental colonisation model has similarities to the situation in humans, in which a decolonisation and recolonisation study indicated that *S*. *aureus* can be acquired by a very large proportion of the human population but that persistent carriers become recolonised for much longer than intermittent or non-carriers^10^.

Our experimental model has some limitations; as we rely on contaminating the environment for mice to become colonised, other possible routes of transmission could occur. For example, *S*. *aureus* could be transmitted to mice by animal handlers within the facility or from contaminated cages. To address this we performed *spa* typing to show that *S*. *aureus* with the same *spa* type is present throughout our long-term colonisation experiments, and included control animals in separate cages which never became *S*. *aureus* colonised. Our experimental model could benefit from further optimisation, to establish the optimum dose, cage type, number of mice per cage and spraying equipment. We have not investigated the impact of microbiota of mice on *S*. *aureus* colonisation, and this would be an interesting area to investigate in future, given that differences in microbiome of pigs which carry *S*. *aureus* versus non-carriers have been observed^21^ and that microbiota of mice can affect *S*. *aureus* susceptibility in pneumonia infection models^22^.

Secondly, we have only established experimental colonisation in one strain of mice (BALB/c) using one *S*. *aureus* strain (SaF_1) and so further work should be done to investigate whether other mouse strains can be colonised, and by other *S*. *aureus* strains such as MRSA strains. The data of Schulz *et al*.^26^ suggests this is likely to be the case. There are other limitations related to using murine models for *S*. *aureus* research, the most striking being that several *S*. *aureus* virulence factors have no effect in mice^44^. However, in light of the ethical considerations for experimental colonisation studies in humans, we feel that our murine experimental colonisation model provides a valuable additional tool for those researching *S*. *aureus* carriage and interventions to reduce it.

This work should allow identification of the acquired factor(s) responsible for control of carriage, including microbiota-*S*. *aureus* interactions, interference with the many proteins involved in *S*. *aureus* adhesion to mucosal surfaces *in vivo* ^45^, and the impact of adaptive immunity on *S*. *aureus* carriage^46^. In addition, our novel experimental *S*. *aureus* colonisation model could be used to investigate ability of SaF_1 mutants in colonising the GI tract^31^ and assess efficacy of vaccine candidates in reducing or eliminating *S*. *aureus* GI colonisation and thus contribute to attempts to eliminate this important pathogen.

## Materials and Methods

### Animals

All mouse procedures were conducted in accordance with the Animal (Scientific Procedures) Act 1986 under a UK Home Office Project licence, and were approved by the University of Oxford Animal Care and Ethical Review Committee. 6 week old female BALB/c mice (both certified *S*. *aureus-*free and colonised) were obtained from Harlan (Blackthorn, UK). Certified *S*. *aureus*-free 6 week old SOPF female BALB/c were obtained from Taconic (Ejby, Denmark). On arrival, animals were randomly distributed into individually ventilated cages, housed in groups of 3, 4, 5 or 6 and fed and watered ad libitum. For neonatal experiments 6 week old BALB/c (*S*. *aureus*-free) male and female mice from Harlan were mated and bred in house. An in-house breeding colony of BALB/c mice originating from Jackson Laboratories, USA prior to 2006 was also investigated.

### *S*. *aureus* identification and carriage monitoring

Stool samples were collected from individual animals to isolate *S*. *aureus* and monitor carriage levels. Stools were weighed, homogenised in 500 μl sterile PBS and plated onto Brilliance Staph 24 (Oxoid Ltd, Basingstoke, UK) agar plates using an Autoplate® Automated Spiral Plater (Advanced Instruments, Inc., Norwood, MA, USA) or by hand. Plates were read using QCount Automated Colony Counter (Advanced Instruments, Inc) or manually after 24 hour incubation at 37 °C. In parallel, homogenised stools were enriched for 24 hours at 37 °C in 5% salt broth (Oxoid Ltd) before being plated onto Brilliance Staph 24 agar plates as above. Suspected positive *S*. *aureus* colonies on Brilliance Staph 24 plates were confirmed using Staphylase Test Kit (Oxoid Ltd).

Cheek and nasal swabbing in mice were performed with a viscose breakpoint swab (Technical Service Consultants Ltd, Heywood, UK) pre-wetted with sterile PBS and rubbed inside each cheek or across the nares. Swabs were streaked onto Brilliance Staph 24 agar and enrichment of swabs was carried out as described above. Nasal washes were performed post mortem in a small number of animals using PBS and *S*. *aureus* recovery determined as above. Distribution of *S*. *aureus* throughout the GI tract was quantified in the same animals by homogenising sections of GI tract in PBS and processing as described above for stool samples.

*S*. *aureus* bacterial recovery from stools and organs was quantified as colony forming units (cfu)/g, from nasal washes as cfu/ml, and from cheek swabs as cfu/swab. The detection limit for stools was estimated as 380 cfu/g (1 cfu/50 µl of average stool mass 0.0263 g in 500 μl PBS) except in Fig. 4 and Supplementary Fig. 2 which was 950cfu/g (1 cfu/20 µl of average stool mass 0.0263 g in 500 μl PBS). Detection limit for cheek swabs was 5 cfu/swab, nasal washes 5 cfu/ml and organs 380 cfu/g. For the purposes of depiction, culture negative samples were plotted below the detection limit (dashed line), at 100 cfu/g, while samples positive upon enrichment only were plotted at the detection limit.

### Genotyping of *S*. *aureus* isolates

#### *spa* typing and resistotyping

*spa* typing of single *S*. *aureus* isolates was performed as previously described^47^, except that PCR products were purified using QIAquick PCR Purification Kit (Qiagen, Manchester, UK) and sequencing was performed by Source Bioscience (Oxford, UK). Resistotyping was performed according to EUCAST recommendations^48^.

#### Whole genome sequencing

DNA was extracted from four isolates, derived from the stools of different mice, which were sequenced using Illumina technology as previously described^49^. Sequences were deposited in the NCBI Short Read Archive with accession numbers SRX1386627, SRX1386628, SRX1386629, and SRX1386631. The isolate referred to as SAF_1 corresponds to SRX1386627.

#### MLST typing from Whole genomic sequencing

*De novo* assemblies were performed using Velvet^50^. Consensus sequence corresponding to *S*. *aureus* MLST loci were extracted using tblastn^51^ from Velvet assemblies and compared with sequences present in PubMLST (http://pubmlst.org) in order to assign a multilocus sequence type (MLST) to the newly identified strain.

#### Phylogeny construction from single nucleotide variants (SNVs)

SNVs were identified across all mapped non-repetitive sites using a previously described approach^52^ involving SAMtools^53^. Mpileup with a consensus of at least 75% was required to support an SNV, and calls were required to be homozygous under a diploid model. Only SNVs supported by at least five reads, including one in each direction, which did not occur at sites with unusual depth, were accepted. Maximum likelihood trees were estimated from the mapped whole genomes, together with a collection of ST15 *S*. *aureus* isolates from a previously described collection representing global *S*. *aureus* diversity^29^ using PhyML^54^ with a Jukes–Cantor model. We examined for evidence of recombination using ClonalFrameML^55^, and estimated likely times of divergence between the 4 isolates from mouse stool and human ST15 strains using BEAST, incorporating sampling dates and fixing the clock rate to 2.72 × 10^−6^, as reported by Young *et al*.^30^, since from the small sample of data available there was limited clock signal, as estimated by root-to-tip regression using Tempest (http://tree.bio.ed.ac.uk/software/tempest/). Having done so, MCMC traces with effective sample size (ESS) > 200 were obtained in each of 4 replicate runs, which all converged to the same distribution.

#### Investigation of gene content

To assess presence/absence of a series of genes, single reads were mapped to selected accessory and core genome gene sequences (Supplementary Table 2) using Bowtie 2 with the –very-sensitive option. We also mapped to control sets comprising RecA and MLST loci (yqil, tpi, pta, gmk, glpf, aroe, arc). Numbers of reads mapping were counted using Samtools^53^. An estimated coverage metric (reads mapped/gene length) was calculated for each gene, and genes were regarded as absent if gene coverage was less than 10% of the median coverage for the control set of genes, using custom R scripts (R version 3.1.1 for Windows).

### Co-housing

Certified *S*. *aureus-*free and colonised BALB/c mice from Harlan were obtained and stool samples were taken as described above to determine baseline carriage levels. Animals were then co-housed; in four cages two animals in each cage were initially *S*. *aureus*-free and two animals were colonised. Two control cages containing only *S*. *aureus*-free or only colonised animals were also included. Stool samples were taken throughout to monitor carriage levels.

### Colonisation by Oral Gavage

BALB/c female mice (Taconic Farms, Denmark) were experimentally colonised with *S*. *aureus* strain termed ‘SaF_1’; an isolate obtained from a stool sample of one of the 4 BALB/c mice (Harlan, UK),which were sequenced as described above. Three to four individual colonies of SaF were picked and cultured overnight in TSB (Tryptic Soy Broth, Oxoid Ltd) at 37 °C with shaking at 130 rpm followed by 1:100 dilution into fresh TSB to culture for 2.5 hours at 37 °C, without shaking. Bacteria were washed and resuspended in PBS. Mice received either SaF (10^8^ cfu in 100 µl PBS) or PBS via an oral feeding tube (Instech, Plymouth, PA, USA) and stool samples were subsequently taken as described above to monitor carriage levels.

### Colonisation by Environmental Contamination

Certified *S*. *aureus-*free BALB/c mice from Harlan were experimentally colonised with *S*. *aureus*. Strain SaF_1 was prepared by overnight culture in TSB (Oxoid) at 37 °C, 130 rpm, washed and resuspended in PBS (Sigma). Contamination of cages was performed by spraying this inoculum onto bedding using a 100 ml plastic spray bottle with a hand pumped vaporiser (product 215–3092, VWR International). Each cage received 5–10 ml of *S*. *aureus* culture at ~5 × 10^9^ cfu/ml. Mice were not sprayed directly, and cages were not cleaned until day 7 after spraying.

### Statistical analysis

Grouped data are presented as means with SEM, and scatterplot data presented with linear regression and 95% confidence intervals (CIs), unless otherwise indicated. Statistical significance of variations in continuous variables by group was analysed by Mann-Whitney or Kruskal-Wallis tests (for skewed data) or ANOVA (for normally distributed data) as stated in results. Comparison of time to endpoint used log-rank (Mantel-Cox) tests. GraphPad Prism software version 6.03 (La Jolla, CA, USA) was used for graphical presentation and statistical analyses of *S*. *aureus* carriage data, while IBM SPSS Statistics 22 was used for other analyses.

## Electronic supplementary material

Supplementary information
