# Supplementary material for: Development of persistent gastrointestinal S. aureus carriage in mice
Source: Sci Rep. 2017 Sep 29;7:12415. doi: 10.1038/s41598-017-12576-0 (PMC5622074; doi:10.1038/s41598-017-12576-0)
Supplement: Supplementary file 1 — Supplementary information [file 41598_2017_12576_MOESM1_ESM.pdf]

Development of persistent gastrointestinal *S. aureus* carriage in mice

Amy Flaxman, Pauline M. van Diemen, Yuko Yamaguchi, Elizabeth Allen, Claudia Lindemann, Christine S. Rollier, Anita Milicic, David H. Wyllie

Supplementary Data

TABLE S1. Successful recovery of *S. aureus* from stool samples, cheek swabs and nasal swabs was compared. *p* values calculated using 2 sided Fisher's Exact test.

Data analyzed	Positive	Negative	Total
Stool sampling	36	0	36
Cheek Swabbing	27	9	36
Total tested	63	9	72
p value	0.002		
Data analyzed	Positive	Negative	Total
Stool Sampling	36	0	36
Nasal Swabbing	1	4	5
Total tested	37	4	41
p value	< 0.001		
Data analyzed	Positive	Negative	Total
Cheek Swabbing	27	9	36
Nasal Swabbing	1	4	5
Total tested	28	13	41
p value	0.028		

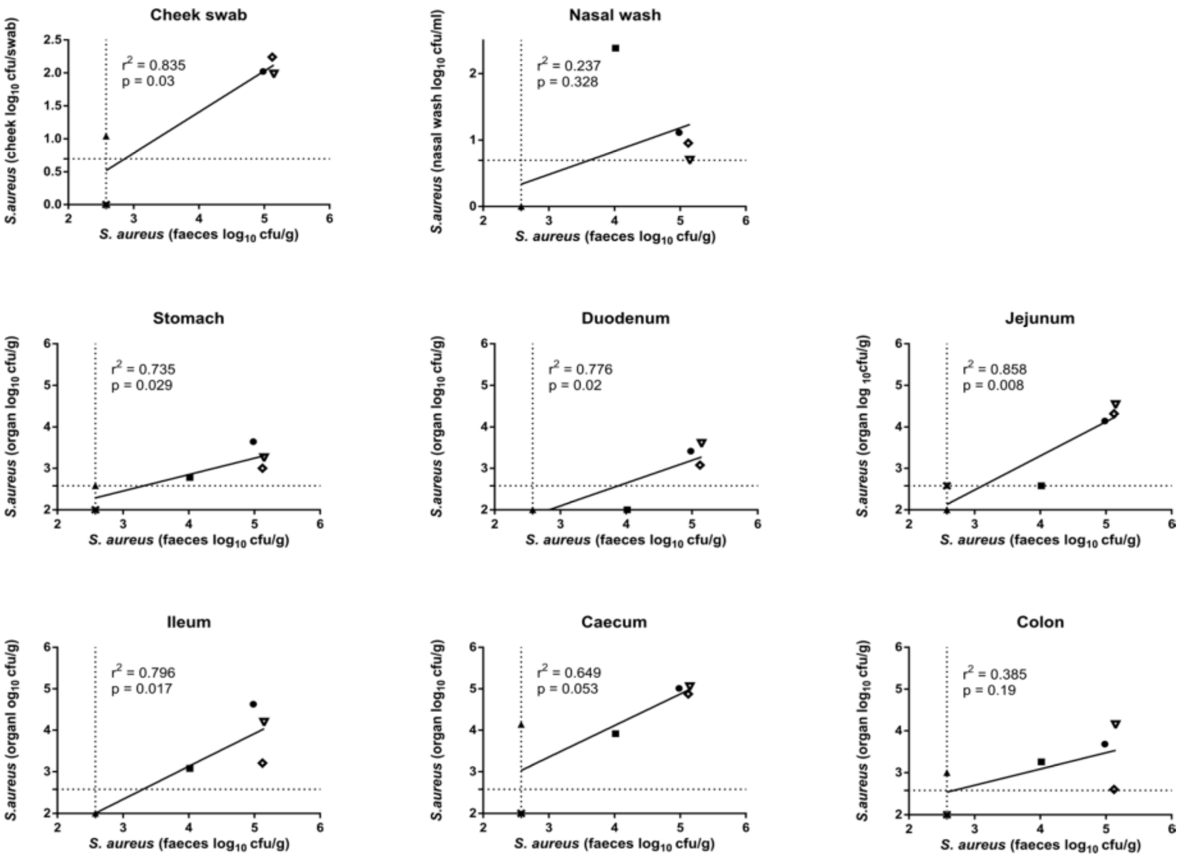

FIG S1 *S. aureus* recovery from throughout GI tract shows positive correlation with recovery from stool samples, as determined by plating onto selective agar. 6 female BALB/c mice from Harlan were confirmed as *S. aureus* carriers on day of arrival by stool sampling. 3 days later stool samples and cheek swabs were taken, animals were culled and organs were removed and nasal wash performed. *S. aureus* recovery from cheek swab, nasal wash and organs against *S. aureus* recovery from stool.

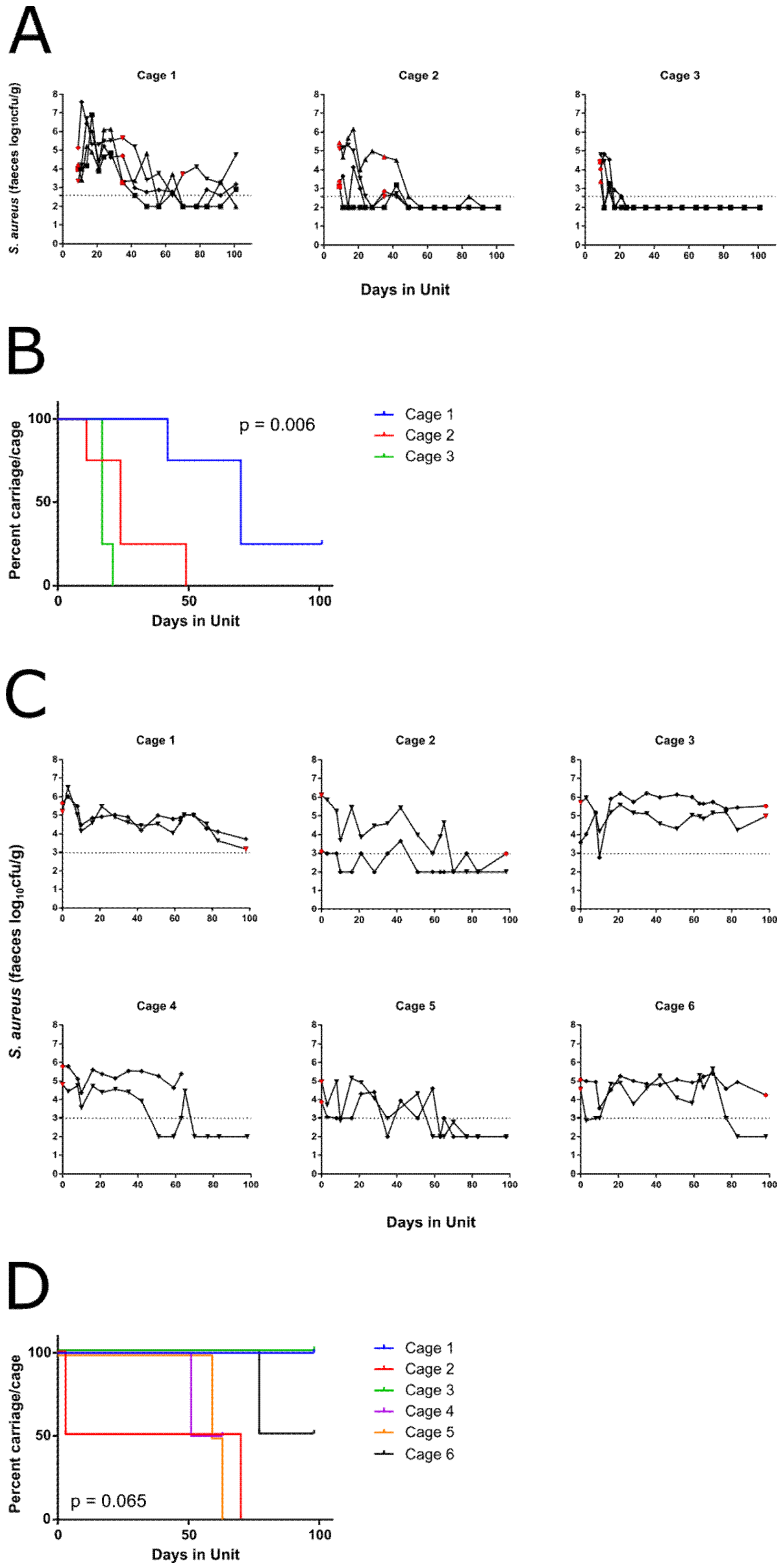

FIG S2 Longitudinal monitoring of SaF carriage in two independent experiments, independent of experiment shown in Figure 3. *S. aureus* carriage levels were monitored by

stool sampling in 12 female BALB/c mice from Harlan, randomly allocated 4 per cage, for 3 months (A & B) and in a further 12 female BALB/c mice from Harlan, randomly allocated 2 per cage, for 3 months (C & D). (A, C) Carriage levels shown per mouse within a cage. Samples coloured red were *spa* typed and found to be t084. In (A) quantitative analysis of carriage was performed from 9 days onwards. (B, D) Kaplan-Meier analysis for loss of carriage per cage. p value given for Log rank (Mantel-Cox) test. *S. aureus* unrelated adverse events occurred as follows – one mouse in Cage 4 had to be culled at 63 days due to illness.

TABLE S2 Targets used to determine gene presence absence in SaF and human CC15 isolates.

>agr type i<br>gtcacaagtactataagctgcgatgttaccaatgtttttaaaatcccagtaataaaatcaaaaaataagttaaataatgtattcattt<br>taagtcctccttaataaagaaaataggtaataatgtaatagcttctattatgatgcctaattgaatgaattgggcaaatggctctttga<br>tgataagtgtgataatgaaaagggttaaactaacaataatcgcataatattttttcgtttaataagtcgcacaggaatgggcttcttt<br>ttagttgctgcaggagcatatactgagattacacctaaagaaataactgttaaaataatcataattaaaaagttaatatgaaaattt<br>actattactaaaggtaaaagtataaatagtataatactttctacataacaccaaaaagaagaaggtgcatgtgcaccatgtgcat
>agr type ii<br>tattactaattgaaaagtggccatagctaagttgtttattgtttccatatgctacacactttctaaaataacttttactatttgtcgtata<br>aattcgttaattcagcgggtactttaggttcatcaaataaactgctgcaagcgtttacgccaccgacaattccgattgctttagctaat<br>ttaattataaaatcaaaaaacatattaacaagtgtattcatgattaatcctccttagggaaaaaaataggtaataatgtaatagattc<br>tactaaaattcctaataacataaattgagcataaaatggatgtattattaaggatagaatcaaaactaataaatacataattataga<br>taaatactttttacgttttactaattttattggtataggttgctttttcgttgctgcaggtgcataaataacaacggaaatcaatcctata<br>atagataaggccagtaaataagtaaaattaatgtctatattaattagaaaataaggtacaaatacaaatgttaaaatactttgtatg<br>taacaaagtatcgaagactttgcatgtgcaccatgtgcat
>agr type iii<br>gtaatgtaatagcttgtataataatacccagttgaataaattgggtatatggttcttttacaaaaagtgtgataatgaaaagaattgt<br>actaattataattgaaaaatatctttttttgctttacaagcctcgctgggataggcttcttcttagtggctgcaggtgcatatttaatcact<br>actccaacacttattaatgctaaaaacatcattagtgtttcattaatatgaaaatgcaatactaatagaggtagcacaataaaaagt<br>gtaatgctttctatataacaccaaaaagatgaaggtgcatgtgc
>agr type iv<br>cgataatgccgtaatacccgtgatatacaaatagtcagatttactgtatttgataccactaataattgatggtactgtaaacattagta<br>ttatttgagttaatacgaataaaacaaaattataactatttaatgattccacttactatcacactttctgtttatattattcgtgtaattg<br>agttaattctttaggtatttcaacttcgtccattatgaaataacatgtactataagaagcaacgttaccaatgtttttaaaacaccagt<br>tataaaatcaaaaaagatgtttaataatgtattcattttaagtcctcctaacaaagaaaataggtaataatgtaatagcttctattatg<br>atgcctaattgcatgaattgagcaaatggttctttgatgataagtgtgataatgaaaaagattagactaacaataatcgcataatttc<br>gtttccgcttaataagtcgcacaggaataggcttctttttagttgctgcaggagcatatctaattatcatacctatggcgataactgtc<br>ataatagtcataattaaaaagttaatatgatagtttactaatattaaaggcaagagtgtgaataaaaaaatactttccacataacac

caaaatgaagaaggtgcatgtgcaccatgtgcat
>chp<br>ataaatttaaatatagaatttaaggagaattaacatcattatgaaaaagaaattagcaacaacagttttagcattaagtttttttaacg<br>gcaggaatcagtacacaccatcattcagcgaaagcttttacttttgaaccgtttcctacaaatgaagaaatagaatcaaataagaaa<br>atgttagagaaagaaaaagcttataaagaatcatttaaaaatagtggtcttcctacaacgctaggaaaattagatgaacgtttgag<br>aaattatttaaagaaaggcacaaaaaaattctgctcaatttgaaaaaatggttattttaactgaaaataaaggttactatacagtatat<br>ctgaatacaccacttgctgaagatagaaaaaatgttgagttactaggtaaaatgtataaaacatacttctttaaaaaaggagagtct<br>aaatcatcttatgtaattaatggtcctggaaaaactaatgaatatgcatactaatagtagttacataaattaaaaggtagatatttctt<br>ttttatataaaggtttggcagacatttcataacttgccaaacctttatatatctaattatcaaactgcactaaactt
>capA<br>atggaaagtacattagaattaacaaaaattaaagaagtattacaaaaaaacttgaagattttaattattttaccgctattatttttaat<br>tattagcgctattgttacatttttcgtcttatcacctaaatatcaagctaatactcaaattttagtgaatcaaactaagggtgacaatcc<br>tcagtttatggcgcaagaggttcaaagtaatattcaacttgtaaatacgtataaagaaattgttaaaagtcctagaattttagatgag<br>gtgtcaaaggacttaaatgataagtattcaccatctaaattgtcgagtatgttgacaattacaaaccaagaaaatacgcaacttatc<br>aacatccaagttaaaagtggtcataaacaagattcggaaaaaattgcgaatagcttcgctaaagttacaagtaaacaaattccgaa<br>gattatgagtgtggataacgtatcaattttatctaaagcagacggtacagcagttaaagtcgcaccaaaaactgtagtgaatctaat<br>cggtgcattctttttaggattagttgtcgcgcttatatatatcttcttcaaagtaattttcgataagcgaattaaagatgaagaagatgt<br>agagaaagaattaggattgcctgtattgggttcaattcaaaaatttaattaa
>CapB<br>ttgctacttatgtcaaaaaaggaaaatacgacaacaacactatttgtatatgaaaaaccaaaatcaacaattagtgaaaagtttcg<br>aggtatacgttcaaacatcatgttttcaaaagcaaatggtgaagtaaagcgcttattggttacttctgaaaagcctggtgcaggtaa<br>aagtacagttgtatcgaatgtagcgattacttatgcacaagcaggctataagacattagttattgatggcgatatgcgtaagccaac<br>acaaaactatatttttaatgagcaaaataataatggactatcaagcttaatcattggtcgaacgactatgtcagaagcaattacgtc<br>gacagaaattgaaaatttagatttgctaacagctggccctgtacctccaaatccatctgagttaattgggtctgaaaggttcaaaga<br>attagttgatctgtttaataaacgttacgacattattattgtcgatacaccgccagttaatactgtgactgatgcacaactatatgcgc<br>gtgctattaaagatagtctgttagtaattgatagtgaaaaaaatgataaaaatgaagttaaaaaagcaaaagcacttatggaaaa<br>agcaggcagtaacattctaggtgtcattttgaacaagacaaaggtcgataaatcttctagttattatcactattatggagatgaataa
>CapC<br>atgattgatattcataaccatatattgcctaatatcgatgacggtccgacaaatgaaacagagatgatggatcttttaaaacaagcg<br>acaacacaaggtgttacagaaatcattgtaacatcacatcacttacatcctcgatataccacacctatagaaaaagtgaaatcatgt<br>ttaaaccatattgaaagcttagaggaagtacaagcactaaatctaaagttttattatggtcaggaaataagaattaccgatcaaatc<br>cttaatgatattgatcgaaaagttattaacggtattaatgattcacgctatttactaatagaatttccatcaaatgaagttccacacta<br>tactgatcaattatttttcgaattacagagtaaaggctttgtaccgattattgcacatccagagcggaataaagcaataagtcaaaac<br>cttgacatactatacgatttaattaacaaaggtgctttaagtcaagtgacaacggcgtcattagcgggtatttccggtaaaaaaatta<br>gaaaattagcaattcaaatgattgaaaacaatctgacacatttcatcggttcagatgcgcataacacagaaatcagaccgttcttaa<br>tgaaagacttatttaatgataagaaattacgtgattattatgaagatatgaacggatttattagtaatgcgaagttagttgttgatgat<br>aaaaaaattcctaaacgaatgccacaacaagattataaacagaaaagatggtttgggttataa
>CapD<br>atgaggggttttatggcacatttatctgtgaaattgcggcttttaatactagcattaatcgattcactgatagtgacattttcagtattc<br>gtaagttattacattttagaaccgtatttcaaaacatattctgtcaaattattaatattggcagctatatcactattcatatcgcatcat<br>atttcagcatttatttttaatatgtatcatcgagcgtgggaatatgccagtgtgagtgaattgattttaattgttaaagctgtgacgaca<br>tctatcgttattacgatggtggtcgtgacaattgttacaggcaatagaccgtttttttagattgtatttaattacttggatgatgcacttga<br>ttttaataggtggctcaaggttattttggcgtatttatcggaaataccttggaggtaagtcatttaataagaagccaactttagttgttg<br>gtgctggtcaagcaggttcaatgctgattagacaaatgttgaaaagtgacgaaatgaaacttgaaccggtattagcagtcgatgat<br>gacgaacataaacgcaatatcacaattactgagggtgtaaaagtccaaggtaaaattgcggatattccagaactagtgaggaaat<br>ataagattaaaaaaatcatcattgcaattccaactattggtcaagagcgtttgaaagaaattaataatatttgccatatggatggcgt<br>tgagttattgaaaatgccaaatatagaagacgtcatgtctggtgagttagaagtgaaccaacttaaaaaagttgaagtagaagattt

actaggcagagatcctgttgaattagatatggatatgatatcaaatgaattgacgaataaaactattttagttacgggtgcaggtggt<br>tcaataggatcagaaatttgtagacaagtttgtaatttctatccagaacgtattattctacttggccatggtgaaaacagtatttattta<br>atcaatcgtgaattgcgaaatcgcttcggaaaaaatgttgatatcgttcctattatagcggatgtgcaaaatagagcgcgtatgtttg<br>aaattatggaaacgtataaaccatacgcagtttatcatgcagcagcacacaagcacgtgccgttaatggaagacaaccctgaaga<br>agcagtacgtaataatattttaggtacgaaaaatactgctgaagctgctaaaaatgcagaggtaaagaaattcgttatgatttctac<br>ggataaagccgttaatccgcctaatgtcatgggagcttcaaagcgaattgcagaaatgattattcaaagtttaaatgatgaaacgca<br>tcgaacaaattttgttgcagtgagatttggtaatgtacttggatcgagaggatctgtgattccacttttcaaaagtcaaattgaagaa<br>ggtgggccagttactgtgacacatcctgaaatgacacgttactttatgacaattcctgaagcttctagactagttttgcaggcagggg<br>cattagcagaaggtggcgaagtatttgtgctagatatgggagaaccagtgaaaattgtagatttggcacgtaatttaattaagctaa<br>gtggtaaaaaagaagacgacatacgcattacttatacagggattagacccggcgaaaaaatgtttgaagagcttatgaataaaga<br>tgaggttcatcctgaacaagtatttgaaaaaatttatcgtggcaaagtacaacatatgaaatgtaatgaagttgaagcgattattca<br>agacatcgtcaatgactttagtaaagaaaaaattattaactatgccaatggcaaaaagggagataattatgttcgatga
>CapE<br>atgttcgatgacaaaattttattaattactgggggcacaggatcattcggtaatgctgttatgaaacagtttttagattctaatattaa<br>agaaattcgtatttttcacgcgatgagaaaaaacaagatgacattcgaaaaaaatataataattcaaaattaaagttctacattgg<br>tgatgtgcgtgatagtcaaagtgtagaaacagcaatgcgagatgttgattacgtattccatgcagcagctttaaaacaagtgccgtc<br>atgtgaattctttccagttgaggcagtgaagacaaatattattggtacagaaaatgtcttacaaagtgctattcatcaaaatgttaaa<br>aaagtcatatgtttatctacagataaggcagcgtatcctattaatgctatgggtatttcaaaagcaatgatggaaaaagtattcgtag<br>ccaaatcaagaaatattcgtagtgaacaaacgcttatttgtggtacaagatacggtaatgtgatggcttcaagaggatcagtaatac<br>ctttgtttatcgacaaaatcaaagctggagaacctttaacgattacagatcctgatatgacaagatttttaatgagcttagaagatgc<br>ggtagaactagttgttcatgcatttaagcatgcagagacaggagatattatggttcaaaaagcaccaagctcaacggtaggggatc<br>ttgcgaccgcattattagaattgtttgaagctgataatgcaattgaaatcattggtacgcgacatggagagaaaaaagcagaaaca<br>ttgttgacgagagaagaatacgcacaatgtgaagatatgggtgattattttagagtgccggcagactccagagatttaaattatagt<br>aattatgttgaaaccggtaacgaaaagattacgcaatcttatgaatataactccgataatacacatattttaacggtggaagagata<br>aaagaaaaacttttaacactagaatatgttagaaacgaattgaatgattataaagcttcaatgagatag
>CapF<br>ttgaatattgtaattacaggagcaaaaggttttgtaggaaaaaacttgaaagcagatttaacttcaacgacagatcatcatattttcg<br>aagtacatcgacaaactaaagaggaagaattagagtcagcattgttgaaagcagactttgtcgtgcatttagcgggtgttaatcgac<br>ctgaacatgacaaagaattcagcttaggaaacgtgagttatttagatcatgtacttgatatattaactagaaatacgaaaaagccag<br>cgatattattatcgtcttcaatacaagcaacacaagataatccttatggtgagagtaagttgcaaggggaacagctattaagagagt<br>atgccgaagagtatggcaatacggtttatatttatcgctggccaaatttattcggcaagtggtgtaagccgaattataactcagtgat<br>agcaacattttgttacaaaattgcacgtaacgaagagattcaagttaatgatcggaatgttgaactaacgctaaactacgtggatga<br>tatcgtcgctgaaataaagcgtgctattgaaggaactccaacgattgaaaatggtgtacctacagtaccaaacgtatttaaagtgac<br>attgggagaaattgtagatttattatacaagttcaaacagtcacgtctcgatcgaacattgccgaaattagataacttgtttgaaaaa<br>gatttgtatagtacgtatttaagctatctacctagtacagactttagttatcccttacttatgaatgtggatgataggggttcttttacag<br>aatttataaaaacaccggatcgtggtcaagtttctgtaaatatttctaaaccaggtattactaaaggtaatcactggcatcatactaa<br>aaacgaaaaatttctagtcgtatcaggtaaaggggtaattcgttttagacatgttaatgatgatgaaatcattgaatattatgtttctg<br>gcgacaaattagaagttgtagacataccagtaggatacacacataatattgaaaatttaggcgacacagatatggtaactattatgt<br>gggtgaatgaaatgtttgatccaaatcagccagatacgtatttcttggaggtatag
>CapG<br>atggaaaaactgaaattaatgacaatagttggtacaaggcctgaaatcattcgtttatcatcaacgattaaagcatgtgatcaatat<br>tttaatcagatattagtacacactggtcaaaattatgattatacattgaatcaaattttctttgatgatttggaattaagacaaccgga<br>ccactacttagaggcagttggaagtaaccttggagaaacgatggggaatattattgcgaagacatatgatgttttattacgcgaaca<br>accagatgcacttttaattcttggtgatacaaatagttgtttagcagcagtatctgctaaacgattaaagattcctgtgttccacatgg<br>aagcgggtaatagatgctttgatcagaatgtacctgaagaaatcaatcgtaaaattgttgaccatgtcagtgatgtgaatctacctta<br>tacggaacatagcagacgttatttattagatgaaggcttcaataaagcgaatatctttgtgacaggatcaccgatgacagaagtgat<br>agaagcgcatcgagataaaattaatcacagtgacgttttaaataaactaggattagaaccgcaacaatacattttagtatctgcgca

tagagaagagaatatcgataatgaaaagaattttaaatcattaatgaatgcgataaatgatattgccaaaaagtataaaatgcctg
tgatttattcaacgcatccaagaagttggaagaaaattgaagaaagtaaatttgaatttgatccattagttaaacagttaaagccatt
tggtttctttgattataatgcattgcaaaaagatgcatttgttgtgctatcagatagtggaacattgtcagaagagtcgtctattttgaa
gttccctggtgtccttattcgaacttccacagaaagaccggaagtactagataaaggtacggttattgtaggtggtattacctataac
aatctaatccaatccgttgaactagcaagagagatgcaaaacaataacgaaccgatgattgatgctattgattataaagacactaa
cgtttcgacaaaggtagttaaaattattcaaagctataaagatattatcaatcgaaatacttggaggaaatga

>CapH
atgaggatagcgattgaaaagataattggtttgctgaaaaaccagtcctctaaagaatcgaatgttaagattcatcgcttggcgtat
attacaaactcaaaatttgatggcaataactatatagatagatggtgtaaaatcaggaattctcacattggtgaatacagttatattg
gatttggtagtgattttaataatgtagaagtaggaagatattgttcgatatcttcggatgtaaaaattgggttaggaaaacatcctac
acactttttagctcatcaccgatttttattctaataataatccatttaacataaagcaaaagtttatagactttaatgaccaaccaag
ccgtacaacaattaaaaatgatgtgtggattggtgcaaatgtaattattatggatggtttaacaataaatactggtgcagtcatagca
gccggctcagttgttactaaaaatgtaggagcatatgaggttgttggtggtgttcctgcaaaagtgattaagaagcgatttgacaata
aaacaattgaaaaacttttggaaagcaagtggtgggagaaaacgcctgacaaactaaaaggattttcggttgaatatttaaataaa
aaggatacttaa

>CapI
atgagaattttaaatattgtatcgagtaatattgttcaagacccaagggtacttaaacaaatagaaacaattaaaggcgttacgaat
gattataaaattgttggaatgaataattcacaagctactaataggcgattggaaaatttagattgtaattatcgtttgttaggtagca
aggtagatcccaaaaatattctttctaaattaattaagcgtataagatttgcaacaggtgttatccgagaaattaaagcttttaaacc
tgacgtgattcatgcaaatgatttcgacgtattattaatggtctatttaagcaattataaaaaagctaatattgtttatgatgcgcatg
aaatatatgcgaaaaatgcctttattaataaagttccacttatttcaaagtttgtagaaagtatagaaaaacacatagtaaaacatc
gtgttaatgccttcgtaacagtaagtcatgcagcaaaagaatattatcaatctaaaggatataagaaggaagcgaatgttattacga
atgcacctattttaaatgatagcagagaatttaaagaaatcgaaaactttaaagaaatcgtatatcaaggtcaaattgtaatggaca
gaggatatgaagagtttattattgcttcatcagcttttaaacaaaatgctccttcattcataattcgagggtttggtccgcatgaagaa
gtgataaaagaactgattagttataactcggaaaatattaggttggataaaccagttgaagtaaaagaattggttgataagttagca
gaaagtaatgttggtgttatcttgacgaaacctgtatctattaattttgaatatacagtatctaataaaatttttgaatgtatacatgct
ggtttaccagtaattttatctcctgtcaaagagcatatttatctcaatgaaaaatataaatttggcattgttttaaaggaagttacgcc
gttagaaattgaaaaggcggttagaaaattaagagataatcacgatttgtttaatcatttacgtcaaaatgcaattaaggcgtctaa
aattttgaattggcaaatagaaagtgaacgattagtagaattatataaattttaa

>CapJ
atgaaatttttttgtactttgtgcaattatcagcatgaacatatttatagtaatctctacatttactaaagaagtattagggttccctata
gagccggtgtattactcaaccatggttggtatagcattaattactacggtgtttgctatttataagataattgtcacgcaagaaattcc
gcgagggttaatattattaattgctatatgtttgctttatctagctttttattatttttcaccagataaggaagagaaactagctaaaaa
taatattctattctttttaacatgggcagttccagcggcaattagtggtatttatattaaatatataaacaaggctacggtagaaagat
tttttaaattagtatttttcatattttctatttcatttatttttgtaattttaataccaaaacttacaggtgagatacctagctatatcaattt
tggacttatgaactatcaaaacgcttcgtacctttcagcatttactgccggattaggcatttatttcattatgaaaggttcagtgaaac
ataagtggatatatgttctatttacaataattgatatccctattgtgtttataccaggagggcgtggaggtgctattttattaattcttta
cggcttatttgcatttatacttattacgtttaaaagaggaatacctattgcagtaaaaagcattatgtatatttttgcattaagcatatc
tagtgtattgatttactttctttttacaaaaggttcgaatactagaacattttcatatctacaaggtggaacacttaatttagaaggtac
ttctggaagaggaccgatttatgaaaaaggtatttactttattcaacaaagtccgttattaggctatgggccatttaactattataaac
taatcggaaatataccacataacatcattattgagttgattctatcatttggcttattagggtttttttatcataatgatttgcattttgcta
ctagtttataaaatgattaggaactatgatccaaacactatagatttactcgttatgtttatagcaatctatccaatcacattattaatg
tttagttcaaattatttagttgtaagtgaattttggtttgtgttgttctattttattacaaaaggacggcgtcatcatggttaa

>CapL
atgaaatttttttgtactttgtgcaattatcagcatgaacatatttatagtaatctctacatttactaaagaagtattagggttccctata
gagccggtgtattactcaaccatggttggtatagcattaattactacggtgtttgctatttataagataattgtcacgcaagaaattcc
gcgagggttaatattattaattgctatatgtttgctttatctagctttttattatttttcaccagataaggaagagaaactagctaaaaa

taatattctattctttttaacatgggcagttccagcggcaattagtggtatttatattaaatatataaacaaggctacggtagaaagat
tttttaaattagtatttttcatattttctatttcatttatttttgtaattttaataccaaaacttacaggtgagatacctagctatatcaattt
tggacttatgaactatcaaaacgcttcgtacctttcagcatttactgccggattaggcatttatttcattatgaaaggttcagtgaaac
ataagtggatatatgttctatttacaataattgatatccctattgtgtttataccaggagggcgtggaggtgctattttattaattcttta
cggcttatttgcatttatacttattacgtttaaaagaggaatacctattgcagtaaaaagcattatgtatatttttgcattaagcatatc
tagtgtattgatttactttctttttacaaaaggttcgaatactagaacattttcatatctacaaggtggaacacttaatttagaaggtac
ttctggaagaggaccgatttatgaaaaaggtatttactttattcaacaaagtccgttattaggctatgggccatttaactattataaac
taatcggaaatataccacataacatcattattgagttgattctatcatttggcttattagggtttttttatcataatgatttgcattttgcta
ctagtttataaaatgattaggaactatgatccaaacactatagatttactcgttatgtttatagcaatctatccaatcacattattaatg
tttagttcaaattatttagttgtaagtgaattttggtttgtgttgttctattttattacaaaaggacggcgtcatcatggttaa

>CapM
atgaagcgattattcgatgtagtgagttcaatatatggtttagtagttttaagtccgattctgttaattacagcattactaattaaaatg
gaatcacctggaccagccattttcaaacaaaaaagaccgacgattaataatgaattgtttaatatttataagtttagatcaatgaaa
atagacacacctaatgttgcaactgatttaatggattcaacatcgtatataacaaagacagggaaggtcattcgtaagacctctatt
gatgaattgccacaattattgaatgttttaaaaggagaaatgtcaattgtaggtcctagaccagcgctttataatcaatacgaattaa
tcgaaaaacgtacaaaagcgaacgtgcatacgattagaccaggtgtgacaggactagctcaagtgatggggagagatgatattac
tgatgatcaaaaagtagcgtatgatcattattacttaacacatcaatctatgatgcttgatatgtatatcatatataaaacaattaaa
aatatcgttacttcagaaggtgtgcatcactaa

>CapN
atgagaaaaaatattttaattacaggcgtacatggatatatcggtaatgctttaaaagataagcttattgaacaaggacatcaagta
gatcaaattaatgttaggaatcaattatggaagtcgacctcgttcaaagattatgatgttttaattcatacagcagctttggttcacaa
caattcacctcaagcaaggctatctgattatatgcaagtgaatatgttgcttacgaaacaattggcacaaaaggctaaagctgaag
acgttaaacaatttatttttatgagtactatggcagtttatggaaaagaaggtcaggttggtaaatcagatcaaattgatacacaaac
accaatgaaccctacgaccaactatggtatttccaaaaagttcgctgaacaagcattacaagagttgattagtgattcgtttaaagt
agcaattgtgagaccaccaatgatttatggtgcacattgcccaggaaatttccaacggttaatgcaattgtcaaagcgactgccaat
cattcccaatattaacaatcagcgcagtgcattatatattaaacatctgacagcatttattgatcaattaatatcattagaagtgaca
ggcgtgtatcatcctcaagatagtttttactttgatacatcgtcagtaatgtatgaaatacgtcgccaatcacatcgtaaaacggtatt
gatcaacatgccttcagtgttaaataagtattttaataagttgtcggtctttagaaaattattcggcaatttaacatacagcaatacgt
tatatgaaaataataatgcacttgaagttattcctggaaaaatgtcacttgttattgcggacatcatggatgaaacgacaaccaaag
ataaggcataa

>CapO
atgaagttaacagtagttggcttaggttatattggtttaccaacatcaattatgtttgcaaagcatggcgtcgatgtgcttggtgttgat
attaatcagcaaacgattgataagttacaaagtggtcaaattagtattgaagaacctgggttacaagaggtttatgaagaggtactg
tcatcgggaaaattgaaggtatctacaacgccagaagcatctgatgtttttatcattgccgttccgacgccgaataatgatgatcagt
accggtcatgtgacatttcgctagttatgcgtgcattagatagtattttaccattttagaaaaagggaatactattattgtagagtcg
acaattgcgcctaaaacgatggatgattttgtaaaaccagtcattgaaaatttaggatttacaataggtgaagatatttgtttagtgc
attgtccagaacgtgtactgccaggaaaaattttagaagaattagttcataacaatcgtatcattggcggtgtgactaaagcttgtat
tgaagcgggtaaatatgtctatcgcacattcgttcagggagaaatgattgaaacagatgcacgtactgctgaaatgagtaagctaat
ggaaaacacatatagagacgtgaatattgctttagctaatgaattaacaaaaatttgcaataacttaaatattaatgtattagatgtg
attgaaatggcaaacaaacatccgcgtgttaatatccatcaacctggtccaggtgtaggcggtcattgtttagctgttgatccgtactt
tattattgctaaagaccctgaaaatgcaaagttaattcaaactggacgtgaaattaataattcaatgccggcctatgttgttgataca
acgaagcaaatcatcaaagcgttgagcgggaataaagtcacagtatttggtttaacttataaaggtgatgttgatgatataagaga
atcgccagcatttgatatttatgagctattaaatcaagaaccagacatagaagtatgtgcttatgatccacatgttgaattagattttg
tggaacatgatatgtcacatgctgtcaaagacgcatcgctagtattgattttaagtgaccactcagaatttaaaaatttatcggacag
tcattttgataaaatgaagcataaagtgatttttgatacaaaaaaatgttgtgaaatcatcatttgaagatgtatcgtattataattatg
gcaatatatttaattttatcgacaaataa

>ClfA

atgaatatgaagaaaaaagaaaaacacgcaattcggaaaaaatcgattggcgtggcttcagtgcttgtaggtacgttaatcggtttt
ggactactcagcagtaaagaagcagatgcaagtgaaaatagtgttacgcaatctgatagcgcaagtaacgaaagcaaaagtaat
gattcaagtagcgttagtgctgcacctaaaacagacgacacaaacgtgagtgatactaaaacatcgtcaaacactaataatggcga
aacgagtgtggcgcaaaatccagcacaacaggaaacgacacaatcatcatcaacaaatgcaactacggaagaaacgccggtaac
tggtgaagctactactacgacaacgaatcaagctaatacaccggcaacaactcaatcaagcaatacaaatgcggaggaattagtg
aatcaaacaagtaatgaaacgacttttaatgatactaatacagtatcatctgtaaattcacctcaaaattctacaaatgcggaaaat
gtttcaacaacgcaagatacttcaactgaagcaacaccttcaaacaatgaatcagctccacagagtacagatgcaagtaataaag
atgtagttaatcaagcggttaatacaagtgcgcctagaatgagagcatttagtttagcggcagtagctgcagatgcaccggcagctg
gcacagatattacgaatcagttgacgaatgtgacagttggtattgactctggtacgactgtgtatccgcaccaagcaggttatgtcaa
actgaattatggtttttcagtgcctaattctgctgttaaaggtgacacattcaaaataactgtacctaaagaattaaacttaaatggtg
taacttcaactgctaaagtgccaccaattatggctggagatcaagtattggcaaatggtgtaatcgatagtgatggtaatgttattta
tacatttacagactatgtaaatactaaagatgatgtaaaagcaactttgaccatgcccgcttatattgaccctgaaaatgttaaaaag
acaggtaatgtgacattggctactggcataggtagtacaacagcaaacaaaacagtattagtagattatgaaaaatatggtaagtt
ttataacttatctattaaaggtacaattgaccaaatcgataaaacaaataatacgtatcgtcagacaatttatgtcaatccaagtgga
gataacgttattgcgccggttttaacaggtaatttaaaaccaaatacggatagtaatgcattaatagatcagcaaaatacaagtatt
aaagtatataaagtagataatgcagctgatttatctgaaagttactttgtgaatccagaaaactttgaggatgtcactaatagtgtga
atattacattcccaaatccaaatcaatataaagtagag

>ClfB
ttactgctgaatcaccatcagcacttccaccaccataacgtacaacattctcattattccaaccgaaaatactgtagtctctatttgtta
caggatcaacattttcttgaataacctgagtttttaagttcttacctgtattgtcgtaatgcccttctactaatactacatatgttttagta
atatcaccaaatttaatactagctacatttggatgctcataatagattctatttttaaattggtctgttacttctttaaggttagagtcat
ttggatctgcatagtagctatctgataatttagatgtatcattcacttcaaaaattctcagttttgtatctgtagcacttactttaccgct
actttcttcgattttatcttggtagcctttaatatacacccacgtattacctaaaactcgttgcttagggttaacaaatactgtttgcttgt
atgtgttttgacctgaagctgtatctacaccaataatttgagaagaaatgttcgcgccatttggtttatcaattcctgcaattggcgaa
ctatagttataagtaattttattattaaacatttcatccgcaatattaatattcgcatcatatgttcctgatttaggtgcctttgctcggtc
tgtaaataaaggtaatgaaaattgtccgttaatattttctttattatttacataatctgtaaagacaaatgtatacgtcttagtcaagat
atcatatgttgctttagctacaacatcgccattcgtacttttaatgtctgcaattggcatcgtattatttgaattagaataatccacgtct
ccattaccagttaaactatctggtaacttcgctgtaaaataatcccctgatttcactttatctgtcactgtaaaatttgccgccataaat
gtgttaccactttgattagggtcaaatgtagtcttttctaacttgaaattacttgccgtaactttatcatttacatttgtacctttagcatc
agcagcatttactaccggttcagcaacagctaaactacgtacagctctcgttctaacacttggtttactagttccttgcgcattggaaa
tcgtttgtggtgatgattgtggtaaatctaatgtttgagaatttttaagctcactgtttgttgctatgctattagcatcattcgttgttttat
tatctacttgagaatttgcttcttgaggaacagtttgatcttgcatttttgcagcagttgcttgatttttaattgccgtcggttgaggtgtt
tcatttgttgaagctggctctgttgtagtggtattgctcgtttgtgtagacattggttttgttgtgctatctacattcgcactgtttgtgttt
gcactaatatcagatgtatcattagccgttgtatttaattgaggtgtttctatcatattgttttttcggaatctgcacttgcattatttttc
gaagattgcgttgtatcgttcgattgttctgaagcttgtgcttgatgattgcctatcccaaatagtatagttgcccctactattactgatg
tggtacctactgtaaaacgtctaatcgaatacttattctgcttattcgacaaataatcaattcttttttcaa

>Hla
atgaaaacacgtatagtcagctcagtaacaacaacactattgctaggttccatattaatgaatcctgtcgctaatgccgcagattctg
atattaatattaaaaccggtactacagatattggaagcaatactacagtaaaaacaggtgatttagtcacttatgataaagaaaatg
gcatgcacaaaaaagtattttatagttttatcgatgataaaaatcacaataaaaaactgctagttattagaacgaaaggtaccattg
ctggtcaatatagagtttatagcgaagaaggtgctaacaaaagtggtttagcctggccttcagcctttaaggtgcagttgcaactacc
tgataatgaagtagctcaaatatctgattactatccaagaaattcgattgatacaaaagagtatatgagtactttaacttatggattc
aacggtaatgttactggtgatgatacaggaaaaattggcggccttattggtgcaaatgtttcgattggtcatacactgaaatatgttc
aacctgatttcaaaacaattttagagagcccaactgataaaaaagtaggctggaaagtgatatttaacaatatggtgaatcaaaat
tggggaccatatgatagagattcttggaacccggtatatggcaatcaacttttcatgaaaactagaaatggttctatgaaagcagca
gataacttccttgatcctaacaaagcaagttctctattatcttcagggttttcaccagacttcgctacagttattactatggatagaaa
agcatccaaacaacaaacaaatatagatgtaatatacgaacgagttcgtgatgattaccaattgcattggacttcaacaaattgga

aaggtaccaatactaaagataaatggacagatcgttcttcagaaagatataaaatcgattgggaaaaagaagaaatgacaaatta<br>a
>hlgA<br>atcggtcaaggtgcagaaatcatcaaaagaacacaagacattactagcaaacgattagctataactcaaaacattcaatttgatttt<br>gtaaaagataaaaaatataacaaagatgccctagttgttaagatgcaaggcttcatcagctctagaacaacatattcagacttaaa<br>aaaatatccatatattaaaagaatgatatggccatttcaatataatatcagtttgaaaacgaaagactctaatgttgatttaatcaat<br>tatcttcctaaaaataaaattgattcagcagatgttagtcagaaattaggctataatatcggcggaaacttccaatcagcgccatca<br>atcggaggcagtggctcattcaactactctaaaacaattagttataatcaaaaaaactatgttactgaagtagaaagtcagaactct<br>aaaggtgttaaatggggagtgaaagcaaattcatttgttacaccg
>hlgB<br>atgaaaatgaataaattagtcaaatcatccgttgctacatctatggcattattattactttctggtactgctaatgctgaaggtaaaat<br>aacaccagtcagcgtaaaaaaagtcgatgacaaagttactttatacaaaacaacagccacagcagattctgataaatttaaaattt<br>cacagattttaacatttaatttcatcaaagataaaagttatgataaagatactttagtacttaaagctactgggaatattaactcagg<br>ctttgtgaaacctaatcctaatgactatgacttttcaaaattatattggggagctaaatacaatgtatctataagctcacaatctaatg<br>attcagtaaacgtcgttgattatgcaccaaaaaatcaaaatgaagagtttcaagttcaaaatactttaggctatacatttggtggtga<br>cattagtatctctaatggtttatctggtggacttaatggaaatacagctttttctgaaacaattaattataaacaagaaagttacagaa<br>caacattaagtcgcaacacaaattataaaaatgttggctggggagttgaagcacataaaattatgaataatggttggggaccttatg<br>gaagagatagcttccacccaacatatggtaatgaactcttcttagctggcagacaaagcagtgcatacgctggccaaaacttcatag<br>cgcaacaccaaatgccattattatctagaagtaacttcaatccagaattttttaagcgtactatcacacagacaagatggcgctaaaa<br>aatctaaaattacagtaacttatcaacgtgaaatggatttataccaaattcgttggaatggcttctactgggcaggcgcaaattataa<br>aaactttaaaactagaacatttaaatcaacatatgaaattgattgggaaaatcacaaagtgaaattgttagatacaaaagaaactg<br>aaaacaataaatag
>HlgC<br>atgcttaaaaataaaatattaactacaactttatctgtgagcttacttgcccctcttgccaatccgttattagaaaatgctaaagctgc<br>taacgatactgaagacatcggtaaaggaagcgatatagaaattatcaaaaggacagaagataaaacaagtaataaatggggcgt<br>gactcaaaatattcaatttgattttgtaaaggataaaaaatataacaaagatgctttgatattaaagatgcaaggattcattagctct<br>agaacaacatattacaactataaaaaaactaatcatgttaaagctatgcgatggccattccaatataatattggtttaaaaacaaat<br>gataaatatgtttctttaattaattatttacctaaaaataaaattgaatctacaaacgtgagtcagacattaggatacaatatcggtg<br>gtaatttccaatcagccccatcactcggtggtaatggatcatttaactattctaaatcgattagctatacacaacaaaattatgtaagt<br>gaagtagaacaacaaaactcaaaaagtgttttatggggcgtcaaagcgaattcattcgccactgaatcaggtcaaaaatcagcctt<br>tgatagcgatttatttgtaggctacaaacctcatagtaaagatcctagagattatttcgttccagacagtgagttaccacctcttgtac<br>aaagtggatttaacccttcatttatcgccacagtatctcatgaaaaaggttcaagcgatacaagcgaatttgaaattacttacggaa<br>gaaacatggatgtcactcatgccattaaaagatcaacgcattatggcaacagttatttagacggacatagagtccataatgcatttg<br>taaatagaaactatactgtgaaatacgaggtcaattggaagactcatgaaatcaaggtgaaaggacagaattga
>IsdB<br>ttagtttttacgttttctaggtaatacgaatgcaacgatgctacttaaagctaataatgccattaatggtaatgtcatatctttatttgat<br>tcttcaccagtttgtggtaatgattttgctttattttcttgtgtatttttattgttttggctttgagtgtgtccatcatttgtgtttttaatgttt<br>gctttttgtaatggagcactatcttttgcttcgctagaacctgctgaagtttgaacaacatcttttgttgtttttgatgaagcagttgttg<br>gttttgcaacattttgagtcgtagatactaccttagttggagttgtactacttgattctacttcacctttagttggttttgtagcaggcgtt<br>ttgtctttacctgactcactagatgcgtcattttctttttcaacacttggtaattgtttattgtcatctttttggctgtcttgtttttgtgattc<br>tttttcaacaggtgatggtgttggtttgctaggcgtagctggagtagcttccttcttagctgagttatcttgttgttctttttttgttagattt<br>atcggtattggcttttgtaaatgcttctttatcaacgattctgacatggtattgtccatcataatcaatcgtttttacgtgaactttaacg<br>atagcatcatatagagttttaccttcaacatatgggaaaataattgttctagtattatttttagcatctttgcttatagttctaacacgtt<br>gaccttcaaccatgaaatctttccagtaatcgtcattagtagtttccatgaccatatattttttgccgttaagcatacctgttttaatagg<br>gtgtttaacaaaagtatccatcatagattcgttattctcaacactttcataaacaacatattttgtatcttgtaaatcagtcatttttttca<br>tttgttggttgtacattttggaattcagtaatagctgatttcacttgctcatctaaagctttctttgtatcctctaatttcttcttgtactca<br>gcctttaatttttcaggaagtttatcttgaattttatttaattcataaacttgtctttctagtgttttcgctttttttatatggcgctaataatt

tttcagctttataatcttcttcagttttgaatttatctgcactgttataaattggttgtgcgaattccattaatgtgtaatcgtatttttcttc<br>tttgttattgaagtgtgttgaactaacaattttaacagcttttgttccgtttgatacagagaagcgaatgtaagcataatctttaacagt<br>atcgtatgatactaatttaattggcaactttttgtcaccttcataaacttcaaattttctccaaaattgacctgattgtaatcctaattca<br>atttctggttttgaatcagtgaaaataactctagcaggtttaacagaacttgcataatgataaaactgttgagttccatctttctttttc<br>atttcaaaatcaattggacgagagtttggtgcgctatgatctttgtcttttattgcagggtttttaatcgcttctctaagttcctgattcaa<br>aataggatatgtattgttagtggcttttgctgctggtttaacttcttttgtttccttaggggctttaacttctttaacttctttagcttctttt<br>gtttcagaagtaggggcctcaacttctttattagatactgagacagcattagctactggtttagtttctggagctttttcagatgttgttg<br>ttggacttgcaactgcttcagtttttggttgtgcttctgtatttgtaccacctgtttcttcagctgctgcttgtgcttcgccatttgacatta<br>ataataaaagtgtactaattgctacagatgcaacgcctagtgatgactttctaattgaataaaatgatttaaattctttttgctgtttgt<br>tcat
>IsdA<br>ttatttagattcttttcttttgaaaagtaataaagataatgaacctaaaagggcaagtgttgcgaaggcaactgtgctaataaagtta<br>tcaactgaagttaaaccagtttttggtaattctttagctttagatgcttgtttaggcgtttcgttatgttttgtaactttgttagtttgttgt<br>gatttattatcacttacagcttgattgttgctttcagatttcgctgttgcaacatctttaacaggtgtttgaactttattttgttcttgagca<br>gtttgtgctgttttaactgtatgagcagtttgtgttttagtttgatcttttgttgtgtcagtacttacaactttagtagagtgattgtcttca<br>actttgcttgttgtagttacagtaggtttaactttttcaactttaggttgaactggtttagtttgctcagtaggtgttttaggttgagctgg<br>ttttggttgaaccggtttaacattgtttggttttgctgcgtcagctaatgtaggaattgctttttcaaattccaaatgcgtagtatatctat<br>gattgtaattaatttgtggcacgacaatatgtactttagtagttaagctcttatatccaggttcaactgcaacattgattgttctagtat<br>ccgctttttttattatcgttaacaacagttgttgctaattcttgattgtttgcattgtaaaatttgtattctttccagaatgatgcattgttta<br>acacggtttggaaataatatttattattttgtttaattactttaccagggtgttgcatatagtcatccatgtgtgacttctctgaagagcc<br>atctttttgcacttggaaattaattggttgtgatgttgcttgagaaacttgtgtgctttgattattagttgcgttcgtagcttctgttgccg<br>cattgacttgttggctgtctgcgcctatgtatacaagggaacctaaaatgatagatgctgtacccattgtaatctttttcatagctgatg<br>aacgttgttctgattgatacttactgtttaaataatgttttgtcat
>IspA<br>ttagtgatccctgctataaaataaatcaacgatttctaataagtgttttgtattgaattgttcatcaatttgcgttagttcatccactgct<br>gcgtctctatgataagtcaatttatcttctgcgccatctttccctaataaactcacgtacgtacttttattattttcaagatcgctgccca<br>ctttttttacctaactttgcttcatcaccatagcagtctaataaatcatctttaatctggaacatcatacctaaatgataactataactttc<br>taaatgttctttagttgtatcatcgacattagcgatatctgctgcactcataaccgcaaaagttaataatgctcctgtttttgttttgtgt<br>atcatttccaaagtttcaagatcaattggttggccttcgctttgcatatctaacatttgaccgccgaccattccaacatgaccacttgct<br>attgacagccgttgtagaacttttatttttacttcatcagttaatctatcatcacttgaaataagttcaaatgctttagttaataaagcat<br>cacctgctaatatcgcagtccactcaccatatactttatgatttgttaattttcctcgtcgataatcatcattatccatcgctggtaggtc<br>atcatgaataagtgaatatgtatgaatcatttctagtgcaattgcgctcttcatacctaactcatactcggtatttagtgaatctaaagt<br>gagtaataacagaactggtcggatgcgtttacctccagcatttaatgaatacaacatactttcttctagctgagtatccattactgatt<br>tatttatcgcaaccgataattcattattgacttcatctattaatttattcatcggtagattcgtcat
>mecA<br>atgaaaaagataaaaattgttccacttattttaatagttgtagttgtcgggtttggtatatatttttatgcttcaaaagataaagaaatt<br>aataatactattgatgcaattgaagataaaaatttcaaacaagtttataaagatagcagttatatttctaaaagcgataatggtgaa<br>gtagaaatgactgaacgtccgataaaaatatataatagtttaggcgttaaagatataaacattcaggatcgtaaaataaaaaaagt<br>atctaaaaataaaaaacgagtagatgctcaatataaaattaaaacaaactacggtaacattgatcgcaacgttcaatttaattttgt<br>taaagaagatggtatgtggaagttagattgggatcatagcgtcattattccaggaatgcagaaagaccaaagcatacatattgaaa<br>atttaaaatcagaacgtggtaaaattttagaccgaaacaatgtggaattggccaatacaggaacagcatatgagataggcatcgtt<br>ccaaagaatgtatctaaaaaagattataaagcaatcgctaaagaactaagtatttctgaagactatatcaaacaacaaatggatca<br>aaattgggtacaagatgataccttcgttccacttaaaaccgttaaaaaaatggatgaatatttaagtgatttcgcaaaaaaatttcat<br>cttacaactaatgaaacaaaaagtcgtaactatcctctagaaaaagcgacttcacatctattaggttatgttggtcccattaactctg<br>aagaattaaaacaaaaagaatataaaggctataaagatgatgcagttattggtaaaaagggactcgaaaaactttacgataaaaa<br>gctccaacatgaagatggctatcgtgtcacaatcgttgacgataatagcaatacaatcgcacatacattaatagagaaaaagaaaa<br>aagatggcaaagatattcaactaactattgatgctaaagttcaaaagagtatttataacaacatgaaaaatgattatggctcaggta

ctgctatccaccctcaaacaggtgaattattagcacttgtaagcacaccttcatatgacgtctatccatttatgtatggcatgagtaac<br>gaagaatataataaattaaccgaagataaaaaagaacctctgctcaacaagttccagattacaacttcaccaggttcaactcaaaa<br>aatattaacagcaatgattgggttaaataacaaaacattagacgataaaacaagttataaaatcgatggtaaaggttggcaaaaa<br>gataaatcttggggtggttacaacgttacaagatatgaagtggtaaatggtaatatcgacttaaaacaagcaatagaatcatcaga<br>taacattttctttgctagagtagcactcgaattaggcagtaagaaatttgaaaaaggcatgaaaaaactaggtgttggtgaagatat<br>accaagtgattatccattttataatgctcaaatttcaaacaaaaatttagataatgaaatattattagctgattcaggttacggacaa<br>ggtgaaatactgattaacccagtacagatcctttcaatctatagcgcattagaaaataatggcaatattaacgcacctcacttattaa<br>aagacacgaaaaacaaagtttggaagaaaaatattatttccaaagaaaatatcaatctattaactgatggtatgcaacaagtcgta<br>aataaaacacataaagaagatatttatagatcttatgcaaacttaattggcaaatccggtactgcagaactcaaaatgaaacaagg<br>agaaactggcagacaaattgggtggtttatatcatatgataaagataatccaaacatgatgatggctattaatgttaaagatgtaca<br>agataaaggaatggctagctacaatgccaaaatctcaggtaaagtgtatgatgagctatatgagaacggtaataaaaaatacgat<br>atagatgaataacaaaacagtgaagcaatccgtaacgatggttgcttcactgttttattatgaattattaataagtgctgttacttctc<br>ccttaaatacaatttcttcattttcattgtatgttgaaagtgaca
>nuc<br>atgaagtcaaataaatcgcttgctatgattgtggtagccatcattattgtaggtgtattagcatttcaatttatgaatcatacgggtcct<br>ttcaaaaggggacgaatcatgaaactgtacaagatttaaatggtaaagataaagtacatgttcaaagagttgtggatggtgatac<br>atttattgcaaatcaaaatggtaaagaaattaaagttaggcttataggggttgatacgccagaaacggtgaaaccgaatacgcctg<br>tacaaccatttggcaaagaagcatcaaattatagtaagaagacattaacaaatcaagatgtttatttagaatatgataaagaaaaa<br>caagatcgctatggtagaacattggcgtatgtatggataagtaaagatcgtatgtacaataaggaattagtggaaaagggacttgc<br>tagagagaagtatttttcaccaaatggcaaatatagaaatgtatttatagaagcacaaaataaagctaaacaacagaaattaaata<br>tttggagtaaataa
>psmA1<br>atgggtatcatcgctggcatcattaaagttatcaaaagcttaatcgaacaattcactggtaaataa
>psmA2<br>atgggtatcattgcaggaatcattaaattcattaaaggattaattgagaaattcactggtaagtaa
>psmA3<br>atggaattcgtagcaaaattattcaaattctttaaagatttacttggtaaatttttaggtaacaactaa
>psmA4<br>atggctattgtaggtactatcattaaaatcatcaaagcaattatcgacattttcgcaaaataa
>psmB1<br>atggaaggtttatttaacgcaattaaagataccgtaactgcagcaattaataatgatggcgcaaaattaggcacaagcattgtgag<br>catcgttgaaaatggcgtaggtttattaggtaaattattcggattctaa
>psmB2<br>atgactggactagcagaagcaatcgcaaatactgtgcaagctgcacaacaacatgatagtgtgaaattaggcacaagtatcgtag<br>acatcgttgctaacggtgtgggtttactaggtaaattatttggattctaa
>eta<br>ctataagagtcagaaaaacagcaggattaaccggcactgtcgttgaagaagattcatggctatacacaaaagatgattgggtaaa<br>attcgatcaagtaattaaaaaagatggctactggtggattagattcaaatatcaacgtgagggctctagtactaacgatttttttgtg<br>cagtatgtagaatcactgacaaggaacaaaagattaaaaatgaaaaatattggggaactattgagtggaattaacaaacgtattta<br>atgtttagttaattaaaagttaataaaaaataatttgttttgaaatagaaacgttatataatttttaatgtattcgaatacattaaaaa<br>acgcaaatgttaggatgattaataatgaataatagtaaaattatttctaaagttttattgtctttatctctatttactgtaggagctagt<br>gcatttgttattcaagacgaactgatgcaaaaaaaccatgcaaaagcagaagtttcagcagaagaaataaaaaaacatgaagag<br>aaatggaataagtactatggtgtcaatgcatttaatttaccaaaagagctttttagtaaagttgatgaaaaagatagacaaaagtat<br>ccatataatactataggtaatgtttttgtaaaaggacaaacaagtgcaactggtgtgttaattggaaaaaatacagttctaacaaat<br>agacatatcgctaaatttgctaatggagatccatctaaagtatcttttagaccttctataaatacagatgataacggtaatactgaaa<br>caccatatggagagtatgaagtcaaagaaatattacaagaaccatttggtgcaggtgttgatttagcattaatcagattaaaaccag<br>atcaaaacggtgtttcattaggcgataaaatatcgccagcaaaaatagggacatctaatgatttaaaagatggagacaaactcgaa

ttaataggctatccattcgatcataaagttaaccaaatgcacagaagtgaaattgagttaacaactttatcaagaggattaagatac<br>tatggatttacagttccgggaaattctggatcaggtatatttaattcaaatggagaattagttggtatacattctagcaaagtgtctca<br>tcttgatagagagcatcaaataaattatggtgttggtattgggaattatgtcaagcgcattataaacgagaaaaatgagtaataaat<br>aaaataaaaatccgtggatgttttatacaaaacttatattttatagcagtaagaagctgactgcatatttaaaccacccatactagtt<br>actgggtggttgtttttttatgttatattataaatgatcaaactacaccacctattaatttaggagtgtggttattttaatatgcgaagct<br>aaaataactacaaatgataccatttttgataccaaaaaataataacctcaaattc
>etb<br>cagtttacttcacagtatgacacattcttactgaattatgttattgggtaacggctatatacattcaattcaatgctatataaataaaat<br>ggatattgtagaatgtgtcatggttattaccactaaaaatagtgaaactaaaattaaaattccgattaataatattattagtgttactt<br>aataaatttataccacctaataccctaataatccaaaaacagaaaatactattaggtatattatcgatggaattataataaataatta<br>tactggagatatttttttgagacagtgcattaaatgaataacttttaattaacttttatttaattaaaagttaataagaattaattaaaa<br>gttaattatacaattaatgtttaatagtataatgtttgtataaaagttaaaaggaggttttatatatggataaaaatatgtttaaaaaa<br>attattttagcagcgtcaatttttactatttccttacctgtgattccttttgaaagtacattacaagcaaaagaatacagcgcagaaga<br>aatcagaaaattaaaacaaaaatttgaggttccacctacagataaagagctttatacacacattacggataatgcaagaagtcctt<br>ataattctgttggtacagtgtttgtcaaaggtagtacattagctaccggagttttaattggtaaaaatacaattgttactaattaccac<br>gttgcaagagaagcagccaaaaacccatcgaatattatttttacacccgctcaaaatagagatgcagaaaaaaatgaattccctac<br>tccgtatggaaaatttgaagctgaagaaattaaagaatctccgtatggacaaggactcgatttagctataataaaattaaaaccaa<br>acgaaaaaggggaatcagcgggagatttaattcaaccagctaatatacctgatcatattgatatacaaaaaggagacaaatattct<br>ttattaggatatccttataattattcagcttactctttatatcaaagtcagattgaaatgttcaatgattctcaatattttggatatactg<br>aggtaggaaactctggatcaggtatatttaatttaaaaggagaattaataggtattcacagtggtaaaggcggacaacataatcttc<br>caataggagtgtttttcaatagaaagataagttcactctattcggttgataatacttttggagacactttggggaacgatttgaaaaa<br>gagagcaaaattagataaataacaaaaatcatttaattgtttaatatttcaatatatttactacgctacaaaaaccatgagttgaacc<br>tctgtgctttttgtacgttaataatttttacaagtcattcaaaaaa
>etd<br>atgactaaaaatatattaaaaaagttatttataacattatctcttgcaatatttctctccccgttgattagtcatgcagaaaataaatta<br>ttcgcaaatacatatgaagaatctgaaattttaaaaaagagagaaaagtacaatgcagccccttccacattaagtgaggaagttttt<br>tcaaaagtttcaaatactatgaaaagcccctataattcagttggaacagtttttattaagggggagactatagcttctggtgtattaat<br>tggcaaaaatacaattataacaaactatcatgtatcaaggatggctaaaaaagatccgactaaagttatattcacacctggttcgac<br>taaaacagaagatggagtatataaaactccatatggacaatttgtagcagaagaaattaatgaacacccatatggacaaggaact<br>gatttgtctataattaaactaaaacccaataaagacggaaagtctgcaggtgatttaattccccctgcaaagattgcagattctatag<br>atttgcaacaaggtgacaaaataagcttgctcggatacccttataacttttctactaattctttatatagaagtgaaattgaaatcttt<br>aatttaaattctggtcaatattttgggtatactgagtcgggaaattctggatccgggttgtttaatttaaaaggagagttagttggtatt<br>catgttggaaaaggaggaaaatataatttaccgataggtaaatttttttaatactgaaattggctcattgtattcagtagataattcatt<br>aagcactttaggtagtgatttaaagaaaagagctgaattacagtctcattag
>lukPV-f<br>atggtcaaaaaaagactattagctgcaacattgtcgttaggaataatcactcctattgctacttcgtttcatgaatctaaagctgataa<br>caatattgagaatattggtgatggcgctgaggtagtcaaaagaacagaagatacaagtagcgataagtgggggtcacacaaaat<br>attcagtttgattttgttaaagataaaaagtataacaaagacgctttgattttaaaaatgcaaggttttatcaattcaaagactactta<br>ttacaattacaaaaacacagatcatataaaagcaatgaggtggcctttccaatacaatattggtctcaaaacaaatgaccccaatgt<br>agatttaataaattatctacctaaaaataaaatagattcagtaaatgttagtcaaacattaggttataacataggtggtaattttaat<br>agtggtccatcaacaggaggtaatggttcatttaattattcaaaaacaattagttataatcaacaaaactatatcagtgaagtagaa<br>cgtcaaaattcaaaaagtgttcaatggggaataaaagctaattcatttatcacatcattaggtaaaatgtctggacatgatccaaatt<br>tatttgttggatataaaccatatagtcaaaatccgagagactattttgttccagacaatgaattacccccattagtacacagtggtttc<br>aatccttcatttattgcaactgtttctcatgaaaaaggctcaggagatacaagtgaatttgaaataacgtatggcagaaatatggatg<br>ttactcatgctactagaagaacaacacactatggcaatagttatttagaaggatctagaatacacaacgcatttgtaaacagaaatt<br>acacagttaaatatgaagtgaactggaaaactcatgaaattaaagtgaaaggacataattga
>lukF

atgaaaatgaataaattagtcaaatcatccgttgctacatctatggcattattattactttctggtactgctaatgctgaaggtaaaat
aacaccagtcagcgtaaaaaaagtcgatgacaaagttactttatacaaaacaacagccacagcagattctgataagtttaaaattt
cacagattttaacatttaatttcatcaaagataaaagttatgataaagatactttagtacttaaagctactgggaatattaactcagg
ctttgtgaaacctaatcctaatgactatgacttttcaaaattatattggggagctaaatacaatgtatctataagctcacaatctaatg
attcagtaaacgctgttgattatgcaccaaaaaatcaaaatgaagagtttcaagttcaaaatactttaggctatacatttggtggtga
cattagtatctctaatggtttatctggtggacttaatggaaatacagctttttctgaaacaattaattataaacaagaaagttacagaa
cattaagtcgcaacacaaattataaaaatgttggctggggagttgaagcacataaaattatgaatggttggggaccttatggaaga
gatagcttccacccaacatatggtaatgaactcttcttagctggcagacaaagcagtgcatacgctggccaaaacttcatagcgcaa
caccaaatgccattattatctagaagtaacttcaatccagaattttaagcgtactatcacacagacaagatcgcgctaaaaaatct
aaaattacagtaacttatcaacgtgaaatggatttataccaaattcgttggaatggcttctactgggcaggcgcaaattataaaaac
tttaaaactagaacatttaaatcaacatatgaaattgattgggaaaatcacaaagtgaaattgttagatacaaaagaaactgaaaa
caataaatag

>lukPV-S
atgaaaaaaatagtcaaatcatcagttgttacatcaattgcattgcttttgctatccaatacagttgatgcagctcaacatatcacacc
tgtaagtgagaaaaaggttgatgataaaattactttgtacaaaacaactgcaacatcagattccgataagttaaaaatttctcagat
tttaacttttaattttattaaagataaaagttatgataaagatacattaatactcaaagctgctggaaacatttattctggctatacaa
agccaaatccaaaagacactattagttctcaattttattggggttctaagtacaacatttcaattaattcagattctaatgactcagta
aacgttgtagattatgcacctaaaaatcaaaatgaagaatttcaagtacaacaaacggtaggttattcttatggtggagatattaat
atctctaacggcttatcaggtggaggtaatggttcaaaatctttttcagagacaattaactataaacaagaaagctatagaactagc
ttagataaaagaactaatttcaaaaaaattggttgggatgttgaagcacataaaattatgaataatggttggggaccatatggcag
agatagttatcattcaacttatggtaatgaaatgtttttaggctcaagacaaagcaacttaaatgctggacaaaacttcttggaatat
cacaaaatgccagtgttatccagaggtaacttcaatccagaatttattggtgtcctatctcgaaaacaaaacgctgcaaaaaaatcg
aaaattactgttacttatcaaagagaaatggatagatatacaaacttttggaatcaacttcactggataggtaataattataaagat
gaaaatagagcaactcatacatcaatttatgaagttgattgggaaaatcatacagttaaattaatagatactcaatctaaggaaaa
aaatcctatgagctaa

>lukS
atgcttaaaaataaaatattagctacaactttatctgtgagcttacttgcccctcttgccaatccgttattagaaaatgccaaagctgc
taacgatactgaagacatcggtaaaggaagcgatatagaaattatcaaaaggacagaagataaaacaagtaataaatggggcgt
gactcaaaatattcaatttgattttgtgaaggatacaaaatataacaaagatgctctgatattaaagatgcaaggattcattagctct
agaacaacatattacaactataaaaaaactaatcatgttaaagctatgcgatggccattccaatataatattggtttaaaaacaaat
gataaatatgtttctttaattaattatttacctaaaaataaaattgaatctacaaacgtgagtcagacattaggatacaatatcggtg
gtaatttccaatcagccccatcactcggtggtaatggatcatttaactattctaaatcgattagctatacacaacaaaattatgtaagt
gaagtagaacaacaaaactcaaaaagtgttttatggggcgtcaaagcgaattcattcgccactgaatcaggtcaaaaatcagcatt
tgatagcgatttatttgtaggctacaaacctcatagtaaagatcctagagattatttcgttccagacagtgagttacctcctcttgtac
aaagtggatttaacccttcatttatcgccacagtatctcatgaaaaaggttcaagcgatacaagcgaatttgaaattacttacggaa
gaaacatggatgtcactcatgccattaaaagatcaacgcattatggcaacagttatttagacggacatagagtccataatgcattcg
taaatagaaactatactgttaaatacgaggtcaattggaagactcatgaaatcaaggtgaaaggacagaattga

>lukE
atgtttaagaaaaaaatgttagctgcaagtttgtcagtaggactgattgcacctttagcatctccgattcaagaatctagagcaaata
ctaatattgaaaatattggtgatggtgctgaagtaatcaaacgtacggaggatgtaagtagtaagaaatggggcgttactcaaaat
gtccaattcgactttgtaaaagataaaaaatataacaaagacgctttaattgttaaaatgcaaggttttattaattccagaacttcatt
ttcggatgtgaagggtagaggatatgaattaactaaacgtctgatttggccattccaatataatataggactgacgactaaagatcc
aaatgttagcttaatcaattcaattaccttacctaaaaccaaaatagaaactactgatgttggtcaaacattaggatataacattgga
ggtaatttccagtcagcaccatctataggtggcaatggctcatttaattattctaaaacaattagttatacccaaaagagttatgtcag
tgaagtagacaagcaaaactcaaaatctgttaaatggggtgttaaagcaaacaaatttgttacccctgatggaaaaaaatttgcgc
atgatagatatttattcgtacaaagtccaaatggtccaacaggttcagcaagagaatattttgctcctgataatcaattgccaccttta
gttcaaagtggctttaatccatcgtttatcactacactatcacatgaaaaaggttcaaagttgatacgagtgaatttgaaattttcata

tggtagaaacttagatattacatatgcgactttattccctagaactggtatttacgcagaaagaaagcataatgcatttgtaaataga<br>aactttgtagttagatataaagttaattggaaaacacacgaaattaaagtgaaaggacataattaa
>lukD<br>atgaaaattgaaaaattaggcaaatcatcagttgcttcatcaattgcactgcttttgctatcgaatacagttgatgcagctcaaaata<br>tcacacctaaaagagagaaaaaagtagatgacaaaatcactttatacaaaacaacagcaacatctgataatgataaattgaatat<br>ttttcaaattttaacgtttaatttcattaaggataaaagttatgacaaagatacgttagtacttaaggcagccggaaacattaattcag<br>gttataaaaattctaatccaaaagattacaattactcacagttttattggggcggtaagtataatgtttcggttagttcagaatcaaat<br>gatgctgtaaatgttgttgactatgcacctaaaaatcaaaatgaagaattccaagttcaacaaacattaggttattcttatggcggag<br>atattaatatatctaatggcttatcaggtggattaaatggatcaaaatcattttcagaaacgataaattataaacaagaaagttaca<br>gaactacgattgatagaaaaacaaatcataaatcaattggctggggtgttgaggcccacaaaattatgaataatggttggggacca<br>tatggtagagatagttatgacccaacatatggtaatgaactgtttttaggcggtgacaaaagtagttcaaatgctggtcaaaatttct<br>tgccaacacatcaaatccctttattggcgcgtggtaactttaacccagaatttataagcgtactttctcataaactttttgatacaaaa<br>aaatctaaaatcaaagtaacttaccaaagagaaatggatagatatactaatcaatggaatcgatcacactgggttggtaataacta<br>caaaaatcaaaatacagtaacgtttacatctacttatgaagttgactggcaaaacatactgttaaaattaatcggtacggattctaa<br>agaaactaatcctggagtataa
>lukM<br>atgtttaagagaaaattattagttacaactttgtcgctaggtctaattgtccctatagctacaccatttcaaggctctaaggctactact<br>aatgcagaagatattggcgacgatgcagaagtgattaaacgtacggaagatgtaagtagtaggaaatggggtgtaacacaaaatg<br>tccaatttgatttcgtaaaagataaaaaatataacaaagacgcattaattattaagatgcaaggttttatcaattctaggacaacttt<br>caatgatgttaaacaaaatagagcaaataaaagaatggtttggccatttcaatataatatcggtcttacatcaaaagaccaaaata<br>cgagcttaatcaattatcttcctaaaaataaaatagaaacagttgatgttggtcaaactttaggatataacattggaggtaaattcca<br>gtcagtaccatctataggcggaaatggatcatttaattattctaagagtattaaatattcccaaaagagttatgtcagcgaagttgaa<br>caacaaagctcaaaaactattaagtgggggttaaagcaaattcttttgttatagcagggcatcgatggtctgcttacgatgaattat<br>tgtttataagaaatacgacaagaggacctaatgctagagactattttgtagacgataatgaattgcccccttttaataacaagtggatt<br>taatccgtcttttatcgcgacagtatctcacgaaaaagattcaggcgatacgagcgaatttgaaattacttacggtagaaatatggat<br>gttacctatgcaacctaccttcctaaacttggtctttatccagaaagaaaacataatgaatttgtaaacagaaactttgtggtcaaat<br>atgaagtgaattggaaaacgtacgaaattaaagtaaaggggcacaactaa
>sea<br>atgaaaaaaacagcatttatactacttttattcattgccctaacgtggacaacaagtccacttgtaaatggtagcgagaaaagcgaa<br>gaaataaatgaaaaagatttgcgaaaaaagtctgaattgcagggaacagctttaggcaatcttaaacaaatctattattacaatga<br>aaaagctaaaactgaaaataaagagagtcacgatcaatttttacagcatactatattgtttaaaggcttttttacaaatcattcatgg<br>tataacgatttattagtagattttgattcaaaggatattgttgataaatataaagggaaaaaagtagacttatatggtgcttattatgg<br>ttatcaatgtgcgggtggtacaccaaacaaaacagcttgcatgtatggtggtgtaacgttacatgataataatcgattgaccgaaga<br>gaaaaaagtgccaatcaatttatggctagacggtaaacaaaatacagtacctttggaaacggttaaaacgaataagaaaaatgta<br>actgttcaggagttggatcttcaagcaagacgttatttacaggaaaaatataatttatataactctgatgtttttgatgggaaggttca<br>gaggggattaatcgtgtttcatacttctacagaaccttcggttaattacgatttatttggtgctcaaggacagaattcaaatacactat<br>taagaatatatagagataataaaacgattaactctgaaaacatgcatattgatatatatttatatacaagttaa
>seb<br>atgtataatagattatttgtttcacgtgtaattttgatattcgcactgatactagttatttatacacccaacgttttagcagaaagccaa<br>ccagatcctaaaccagatgagttgcacaaagcgagtaaattcactggtttgatggaaaatatgaaagttttatacgatgataatcat<br>gtatcagcaataaacgttaaatctatagatcaatttctatactttgacttaatatattctattaaggacactaagttagggaattatga<br>taatgttcgagtcgaatttaaaaacaaagatttagctgataaatacaaagataaatacgtagatgtgtttggagctaattattattat<br>caatgttatttttctgaaaaaacgaatgatattaattcacatcaaactgataaacgaaaaacttgtatgtatggtggtgtaactgagc<br>ataatggaaaccatttagataaatatagaagtattactgttagggtatttgaagatggtaaaaatttattgtcttttgacgtacaaact<br>aataagaaaaaagtgactgctcaagaattagattacctaactcgtcactatttggtgaaaaataaaaaactctatgaatttaacaac<br>tcaccttatgaaacgggatatattaaatttatagaaagtgagaatagcttttggtatgacatgatgcctgcaccaggagataaatttg<br>accaatctaaatatttaatgatgtacaatgataataaattggttgattctaaagatgtgaagattgaagtttatcttacgacaaagaa

aaagtga
>sec<br>ataaaaaatgtataagagattatttatttcacgtgtaattttgatattcgcactgatactagttatttctacacccaacgttttagcaga<br>gagtcaaccagaccctatgccagatgatttgcacaaatcaagtgagtttactggtacgatgggtaatatgaaatatttatatgatgat<br>cattatgtatcagcaactaaagttaagtctgtagataaatttttggcacatgatttaatttataacattagtgataaaaaactgaaaa<br>attatgacaaagtgaaaacagagttattaaatgaagatttagcaaagaagtacaaagatgaagtagttgatgtgtatggatcaaat<br>tactatgtaaactgctatttttcatccaaagataatgtaggtaaagttacaggtggtaaaacttgtatgtatggaggaataacaaaac<br>atgaaggaaaccactttgataatgggaacttacaaaatgtacttgtaagagtttatgaaaataaaagaaacacaatttcttttgaag<br>tgcaaactgataagaaaagtgtaacagctcaagaactagacataaaagctaggaatttttaattaataaaaaaaatttgtatgag<br>tttaacagttcaccatatgaaacaggatatataaaatttattgaaaataacggcaatactttttggtatgatatgatgcctgcaccag<br>gcgataagtttgaccaatctaaatatttaatgatgtacaacgacaataaaacggttgattctaaaagtgtgaagatagaagtccacc<br>ttacaacaaagaatggataatgttaatccgattttgatataaaaagtgaaagtattagatattgnaag
>sed<br>gatcaaatatattgatataatgaaagtgagcaagttggatagattgcggctagtctcttgaaaaaagggattcctatgtttatagttg<br>gtacttctaggaaggactagcatgtcttatttcgaaatgctgatggttgtattaacaattatgagtttagtcttaattagtaatcaaaac<br>tctgaatgtattgtctacataatcggtgtaacaataatatatatccgaaagattaaagtactctataaaagaatcatttaaaggtgct<br>caaatttattgaaaaaaggtggaatatgaaaaaatttaacattcttattgcattactctttttttactagtttggtaatatctcctttaaac<br>gttaaagccaatgaaaacattgattcagtaaaagagaaagaattgcataaaaaatctgaattaagtagtaccgcgctaaataatat<br>gaaacattcttatgcagataaaaatccaataataggagaaaataaaagtacaggagatcaatttttagaaaatactttgctttacaa<br>aaaatttttttactgaccttatcaattttgaagatttattaataaacttcaattcaaaagaaatggctcaacatttcaaatctaaaaatg<br>tagatgtttaccctataagatatagcattaattgttatggtggtgaaatagataggactgcttgtacatatggaggtgtcactccacac<br>gaaggtaataaattaaaagaacgaaaaaaaataccaatcaatttgtggataaatggtgtacaaaaagaagtttctttagataaagt<br>tcaaacagataaaaaaaatgttaccgtacaagaattagatgcacaagcaaggcgctatttgcaaaaggatttaaaattgtataata<br>atgatactctcggaggaaaaatacagcgcggaaaaatagagtttgattcttctgatgggtctaaagtctcttatgatttatttgatgtt<br>aagggtgattttcccgaaaaacaattacgaatatacagtgataataaaacattatccacagagcaccttcatattgacatctatttat<br>atgaaaagtagtcaattcaaatactttaacatcagacactaaagccatttcaaagaatgctaaagatttattaatcggt
>see<br>atgaaaaaaacagcatttatactacttttattcattgccctaacgttgacaacaagtccacttgtaaatggtagcgagaaaagcgaa<br>gaaataaatgaaaaagatttgcgaaaaaagtctgaattacaaagaaatgctttaagcaatcttaggcaaatttattattataatgaa<br>aaagctataactgaaaacaaagagagtgatgatcagtttttagagaatactttgttatttaaaggtttttttcacaggtcatccatggta<br>taacgatttattagtagatcttggttcaaaagatgctactaataaatataaagggaaaaaagtagacttatatggtgcttattatgga<br>tatcaatgtgctggaggcacaccaaataaaacagcatgtatgtacgggggtgtaacattacatgataataaccgattgaccgaaga<br>aaaaaaagtaccaattaacttgtggatagacggaaaacaaactacagtacctatagataaagttaaaacaagcaaaaaagaagt<br>aactgttcaagagctagatcttcaggcaaggcattatttacacggaaaatttggtttatataactcagacagctttggcggtaaggtg<br>caaagaggcttgattgtgtttcattcttctgaagggtccacggtaagttatgatttgtttgatgctcaagggcaatatccagatacatt<br>attaagaatttacagagataataaaactattaattcagagaacctccatattgatttgtatttatacacaacttga
>seg<br>tgaatttttagttttaaaacaatgctatcgacacactacaacctgaactatctataagcgtgaatttataataaggttcattgtcaaat<br>agactgaataagttagaggaggttttatgaagaaattatctactgtaattattattttgattctagaaatagtttttcataatatgaatt<br>atgtgaatgctcaacccgatcctaaattagacgaactaaataaagtaagtgattataaaaataataagggaactatgggtaatgta<br>atgaatctttatacgtctccacctgttgaaggaagaggagttattaattctagacagtttttatctcatgatttaatttttccaattgagt<br>ataagagttataatgaggttaaaactgaattagaaaatacagaattagctaacaattataaagataaaaaagtagacatttttggc<br>gttccatatttttatacatgtataatacctaaatctgaaccggatataaaccaaaattttggaggttgttgtatgtatggtggtcttaca<br>tttaatagttcagaaaatgaaagagataaattaattactgtacaggtaacaatcgacaatagacaatcacttggatttacaataact<br>acaaataagaatatggttactattcaggaactagattacaaagcaagacactggctcactaaagaaaaaaagctatacgagtttga<br>tggttctgcatttgaatctggatatataaaatttactgaaaagaacaatacaagtttttggtttgacttatttcctaaaaaagaactag<br>tacctttttgttccatataagtttttaaatatttacggagataataaagttgttgattctaagagtattaaaatggaagtatttcttaatac

tcactgataagtatatttgatatcactatacaatcataaagtgcgtttttcgaacttttgttctaagtgttttatagattcatgtaaatag<br>ttattttattgccagttgaattatttaattttt
>seh<br>attaaactatcatttccattctaactactatagcaactgatatctatgaatataaatcttttaatattggtttaatataaatattccattt<br>ctagaaacctatattttatgtttgtataaattcataaattgttcgaaatttatatattaaaagttgtttttgttgttttataattaagttaat<br>gaaatatattgaggagttttaaaatgattaataaaattaaaatattattttcgtttttagcattattactttcattcacatcatatgcgaa<br>agcagaagatttacacgataaaagtgagttaacagatttagctttagctaatgcatatggtcaatataatcacccattcattaaaga<br>aaatataaagagtgatgaaataagtggagaaaaagatttaatatttagaaatcaaggtgatagtggcaatgatttgagagtaaagt<br>ttgcaactgctgatttagctcagaagtttaaaaataaaaatgtagatatatatggggcatctttttattataagtgtgaaaaaataag<br>tgaaaatatttctgaatgtctatatggaggtacaacactaaatagtgaaaaattggcacaggaaagggtgattggtgctaatgtttg<br>ggtagatggtattcaaaaagaaacagaattaatacgaacaaataagaaaaatgtgacattgcaagaattagatataaagatcaga<br>aaaatattgtccgataaatataaaatttattataaagacagcgaaataagtaaaggtctaattgaatttgatatgaaaactcctaga<br>gattactcattcgacatttatgatttaaaaggagaaaatgactatgagatagataaaatttatgaagacaataaaactttaaaatct<br>gatgatataagtcatattgatgtaaatctatatactaagaaaaaagtataatcaatgaaaatttctagatca
>sei<br>ataatgacaacaaaactgtcgaaactgaaaaattccatttagatgtagaaatatcttataaggacgaaagttgaagtatttcaatca<br>taacttagtaaaggaaatgccatgaaaaaatttaaatatagttttatattagtttttatattactttttaacattaaagatcttacgtat<br>gctcaaggtgatattggtgtaggtaacttaagaaatttctatacaaaacatgattatatagatttaaaaggcgtcacagataaaaac<br>ctacctattgcaaatcaactcgaattttcaacaggtaccaatgatttgatctcagaatctaataattgggacgaaataagtaaattta<br>aaggaaagaaactggatatttttggcattgattataatggtccttgtaaatctaaatacatgtatggaggggccactttatcaggaca<br>atacttaaattctgctagaaaaatccctattaatctttgggttaatggcaaacataaaacaatttctactgacaaaatagcaactaat<br>aaaaaactagtaacagctcaagaaattgatgttaaattaagaagatatcttcaagaagaatacaatatatatggtcataataacac<br>tggtaaaggcaaagaatatggatataaatctaaattttattcaggttttaataatgggaaagttttatttcatttaaataatgaaaaat<br>cattttcatatgatttgttttatacaggagatggactgcctgtaagtttttttgaaaatttatgaagataataaaataatagaatctgaa<br>aaatttcatcttgatgtcgaaatatcatatgtagatagtaactaatgataattagttttaacactaaaatgcgaatttatacgaaatta<br>atatatgtattaactatttaaattttaaagtctctagggctaatgtgtacgttaaatattccctgt
>sej<br>atgaaaaaaacaatatttatactgattttctccctgacgttaacactactaataacccctttagtttacagcgatagcaaaaatgaaa<br>caattaaagaaaagaatttgcacaaaaaatcagagttaagtagtattactttaaataacctcagacatatatacttctttaacgaaa<br>agggtatctctgaaaagataatgacagaagatcaattttttagattacacactattatttaaaagtttttttcataagtcattcacagtat<br>aacgacttattggtacaattcgattcaaaagaaacagttaataagtttaaaggcaaacaagtagatctatacggatcttattatggtt<br>ttcaatgttctggtggtaaaccaaataaaacagcatgtatgtatggtggagtaacactgcatgaaaacaatcaactttatgatacta<br>aaaaaatacctattaatttatggattgatagcatcagaactgttgttccgctagatatagttaaaacaaataagaaaaaagtaacca<br>ttcaagaactagatcttcaggcaagatattatttacataaacaatataatttatacaaccctagtacctttgatggtaaaattcagaa<br>aggactaatcgtgtttcatacctcaaaagaaccgttggttagttatgatttatttaatgttataggtcaatatccagacaaattattaa<br>aaatataccaagataataaaatcatagaatctgaaaacatgcatatcgatatatatttatatacaagcctaattgtattaataagtct<br>acctttggttctgtag
>seu<br>tgaaattaacatatgtattaactatttaaattttaatgcctttaatggtaatgtgtacgtttaatattacctgtaatacatcgaatttata<br>aaaatttaaaatatggagttgttggaatgaagttatttgcttttatcttcatatgtgttaagtcttgcagcttactatttatgttaaatgg<br>caatcctaaaccagaacaattgaataaagcgagtgaattcactggtctaatggataatatgaggtatttgtatgatgataaacacgt<br>atcagaaataaacattaaagcccaagagaagtttttacaacatgatttattatttaaaataaatggctctaaaattgatggttctaaa<br>attttaaaaacagaatttaataataatagcctttcggataaatacaaaaataaaaacatagatttgtttgggacaaactattattatc<br>aatgctatttttcagcggataatatggaattaaatgatggtagactaattgaaaaaacgtgtatgtatggcggtgtgaccgagcatg<br>atggaaatcaaatagataaaaataattcaactgataactctcataatatcttaattaaagtttttgaaaacgagagaaattcattatc<br>ttttgatatacctactaataagaaaaacataacagcacaagaaatagattataaagttagaaactatttacttaagcataaaaattt<br>atatgaatttaatagttcgccttatgagactggctatataaagtttatcgaaggaaatggtcattctttttggtatgatatgatgcctga

atctggtgaaaaattttatccgactaaatatttactaatttataatgataataagacagttgagagtaaatctattaatgtagaagttc<br>atttaaccaaaaaataattgagggagattatattataaaaaatattaaaaagtttatgagattgttctgcatagctgcgattataata<br>actttattatgtcttataaataataattatgttaatgctgatgtagac
>selR<br>atgttaaataaaatcttacttttattgttctctgttacttttatgttgttatttttctctttacattcagtaagtgctaaaccagatccaagg<br>cctggagaattgaatagagttagtgattataaaaaaaacaaaggtactatggggaatgttgaatccttatataaggataaagcggt<br>aatagcagaaaatgttaaaaatactagacaattcttaggacatgatttgatttttcctattccttattcagaatacaaagaagttaaa<br>agcgaatttataaataaaaaaacagctgataaatttaaagacaaacggttagatgtgtttggaataccctatttttatacatgtttag<br>tacctaagaatgaatcaagagaagaatttatttttgatggagtttgcatatacgggggcgttacaatgcactccacagctgatagtat<br>atccaaaaatataattgttccagttactgtagataataaacaacagttctcttttacaattagtacaaataaaaaaacggttacggta<br>caagagctagattataaagtaaggaattggttgactaacaataaaaaattatatgaatttgatgggtcggcatatgagacgggttat<br>atcaaatttattgaacaaaataaagatagtttttggtacgatttgtttcctaaaaaggatttggttccctttataccttataaatttgtaa<br>atatttatggagataataaaactatagatgcttcgagtgttaagatagaagttcacctgactacaatgtga
>sep<br>gtaaataatgatcattccataattattgtatataactaataattactccaacaaaaataatctattatacaaaaatttcggataataac<br>aagtttatatggaattatgctttagaggtgagtaaaataaaaaaaacaacatttatactactttcatttattgccctaacgttgataac<br>aagtccatttgtaaattgtagcgagaaaagcgaagaaataaatggaaaagatttacaaaaaaagtctgaattgcagggaactgct<br>ttaagcaatcttagacaaacctattatcataatggaagtgctataattgaaaataaagagagtaatgatcaatttttaaagaacact<br>atattgtttaatgatttttttacaggtcatcaatggtataatgatttattagtagaccttggttcaaaagacaccgccaatatatacaaa<br>gggaaaaaagtggatttatatggtgtttattatggttatcaatgtactgggggtacaccattcaaaacagcttgtatgtatggcggtgt<br>aacgttacatgataataatcaattggaggaagaaaagaaagtaccgattaacttatggatagatggtaaacaaaatacagtacctt<br>tgggaacggttaaaactaataaaaaagaagtaactgttcaggagctagaccttcagtcaagacattatttacatgaaacatataatt<br>tatataacacagatgcatttaatggaaaaatacaaagaggattaattgagtttcacccttcttctggtgattcggttggttatgatttat<br>ttggtgctcaaggacaatatccagatacacagttgaggatatatagagataataaaacgattaagtctaaaaatatgcatattgata<br>tatatttatatacaacttgagtgaaattttggctgaaaaaatttaaaatctataaaaagtaccataagttattttattactatacttaaa<br>acattcactttttactgctgtttgtaagttttataaaaaatttctat
>tsst-1<br>tatatatttaaacaatagtttagagatggttaattgattcatttaaataatatttatacattctatgcgtaaacgtttacacatttaaatg<br>aaggagaattaaaaatgaataaaaaattactaatgaaatttttcatcgtaagccctttgttgcttgcgacaatcgctacaggttttac<br>ccctgttcccttatcatctaatcaaataatcaaaactgcaaaagcatctacaaacgataatataaaggatttgctagactggtatagt<br>agtgggtctgacgcttttacaaatagtgaagttttagataattccttaggatctatgcgtataaaaaacacagatggcagcatcagcc<br>ttataatttttccgagtccttattatagccctactttttcaaaaggggaaaaagttgacttaaacacaaaaagaactaaaaaaagcc<br>aacatactagcgaaggaacttggattcatttccaaataagtggtgttacaaatactgaaaaattacctactccaatagaactaccttt<br>aaaagttaaggttcatggtaaagatagtccattaaagtattggccaaagttcgataaaaaacaattagctatatcaactttagacttt<br>gaaattcgtcatcagctaactcaaatacatggattatatcgttcaagcgataaaacgggtggttattggaaaataacaatgaatgac<br>ggatccacatatcaaagtgatttatctaaaaagtttgaatacaatactgaaaaaccacctataaatattgatgaaataaaaactata<br>gaagcagaaattaattaatttaccactttttctgtaataattattaataaagggagtccattaattataataagtgttctcccttttatct<br>ttataacgctttaatataaagctagggtgaatta
>chp<br>tcattttaggaatttcgcaaaactaactataaatttaaatatagaatttaaggagaattaacatcattatgaaaaagaaattagcaa<br>caacagttttagcattaagtttttaacggcaggaatcagtacacaccatcattcagcgaaagcttttacttttgaaccgtttcctaca<br>aatgaagaaatagaatcaaataagaaaatgttagagaaagaaaaagcttataaagaatcatttaaaaatagtggtcttcctacaa<br>cgctaggaaaattagatgaacgtttgagaaattatttaaagaaaggcacaaaaaaattctgctcaatttgaaaaaatggttattttaa<br>ctgaaaataaaggttactatacagtatatctgaatacaccacttgctgaagatagaaaaaatgttgagttactaggtaaaatgtata<br>aaacatacttctttaaaaaaggagagtctaaatcatcttatgtaattaatggtcctggaaaaactaatgaatatgcatactaatagta<br>gttacataaattaaaaggtagatatttctttttatataaaggtttggcagacatttcata
>sak

tcatcttaaataagggtgttagctataaaagagataaataaaacaaatatattatatttggaggaagcgccatgctcaaaagaa
gtttattatttttaactgttttattgttattattctcattttcttcaattactaatgaggtaagtgcatcaagttcattcgacaaaggaaaa
tataaaaaaggcgatgacgcgagttattttgaaccaacaggcccgtatttgatggtaaatgtgactggagttgatggtaaaggaaat
gaattgctatcccctcattatgtcgagtttcctattaaacctgggactacacttacaaaagaaaaaattgaatactatgtcgaatggg
cattagatgcgacagcatataaagagtttagagtagttgaattagatccaagcgcaaagatcgaagtcacttattatgataagaata
agaaaaaagaagaaacgaagtctttccctataacagaaaaaggttttgttgtcccagatttatcagagcatattaaaaaccctggat
tcaacttaattacaaaggttgttatagaaaagaaataaaacaaaatagttgtttattatagaaagcaatgtcttgattgaatatgtgt
agtgaaaattatctttcatcaaatt

>scn
ttttagttagtcattaaaataaattagtaccaattactaaggagaataaaaaatgaaaattagaaaatctatacttgcgggaacttta
gcaatcgttttagcatcaccactagtaactaatctagataaaaatgaggcacaagctagcacaagcttgccaacatcgaatgaata
tcaaaacgaaaagttagctaatgaattaaaatcgttattagatgaactaaatgttaatgaattagctactggaagtttaaacacttat
tataagcgaactataaaaatttcaggtctaaaagcaatgtatgctcttaagtcaaaagactttaagaaaatgtcagaagcaaaata
tcaacttcaaaagatttataacgaaattgacgaagcactaaaaagtaaatattaaaaaaaccacccttttacgggtggttttaatttt
ctagataatataaaagtgttc

>psm-mec
ttaaccgaaagcctgaatgcaagtcttgattaaatcaataatgcttgtaataacaccagtgaaatccat

>eap
ttagaatttaagttcaatatctaccttttaatgtcagttgctttaaatactctttcatctttatactcagtttttaagtctacatattttga
cgaaccatttttaaaatacactttatacactgcgcgttcagcaagttctatgtcatgtttagttatcccttgatcatttgcaattgctgat
ttaagttttatacttaaatcattaattgtaattttgcttgcattatagaacacaaattcatttgaaactatgttttcaaatttatttattgtt
actgggaatttcacattattagatttatttattttttttgatttagtgtattgttttacatcaatatctatctttttaatatctttggcacta
aataaattaggtgtaaagatgtctgattttaaattcactacttgtttctttccatttttgaaatatactgtatattttgcttgtttcgcaaa
ttttaagtcaagatcactgatgcctctttcactttttaatacagatttcactttgtcatttaaatatttgtaactaattaattgtttattcga
aatttttagttttgataaaattggtgttgatgtgccatttactgcaattgtatatggtacatagctatccgctttcgcttttgaccctgtttt
aacatcaatatctatctttttaatatctttggcactaaataaattaggtgtaaagatgtctgattttaaattcactacttgtttctttccat
ttttgaaatatactgtatattttgcttgtttcgcaaattttaagtcaagatcactgatacctctttcactttttaatacagatttcactttgt
cgtttaaatatttgtaactaattaattgtttattcgaaatttttagttttgataaaattggtgttgatgtgccatttactgcaattgtgtat
ggtacatatctgtctgctttcgctttagttcctgttttaacagtaatttcaattctcttaacatctttcgcatgaactaagtttgctgtgta
attttttgaatttaactgtaagattcttttaccaccatttttaaaataaacagtataataagctttcttagcatattttaaatcttgttcac
taatacctctatcatgcttcagtactgattttatttgactagttaaatttttgtaatttgtccaaggtttatttgaaaatgaaagattcgat
aaaatgtttgtagatgtaccatttagattaattgaatatggaacttgatagtttcttttagctttattttcgatatgttttttgtatctacg
ttaatattgatacttttaatatcacttgaattgattaaattcgctgtgtaaatacctgatttcaaatctataactttcttcgttccattttta
aaattaactgtatatttcgcttgcttcgaaagtcttaaatcaacatcagtaatacctctatttgattctaaaactgatttaactttaccct
ctaaatctttgtaactaatattttgatttttattaaatgttaggtttgataaaatgttttggcttgtgccgttcactgtgattgtatatggc
acttgaacatttgctttagctttatctttaggttgctttttagtatctacgttaacactgatagctttaatgtcacttgtattgattaagtca
gctgtgtagatacctgatttcaaatcgataactctttttgttccatttttaaaatgaaccgtatattccgcttgctttgaaagtcttaaat
cgatatcactaatacctctattaaagtataaaactgatttaactttattctctatatctttataactaatattttgattcttattaaatgtt
aagcttgataaaatgttttggcttgtgccgttcacagtaattgtatatggaatctggatgttagaatgtccatggtgtaatgtacttgat
gatttatctaatggcttagctgcggcagatgcttcgttagtattaaagtttgctcctgttgatgctataacgcctaatgctaatgttgttg
taatcaatgacttaaatttcat

>hlb
gggcaatataaacgcgctgatttaatcggacaatcttcttatattaaaaataatgatgtcgtaatattcaatgaagcatttgataatg
gtgcatcagacaaattattaagtaatgtgaaaaaagaatatccttatcaaacacctgtactcggccgttctcaatcagggtgggaca
aaactgaaggtagctactcatcaactgttgcagaagatggtggcgtagcgattgtaagtaaatatcctattaaagaaaaaatccag
catgttttcaaaagcggttgtggattcgataatgatagcaacaaaggctttgtttatacaaaaatagagaaaaatggtaagaacgtt
cacgttatcggtacacatacacaatctgaagattcacgttgtggtgctggacatgatcgaaaaattagagctgaacaaatgaaaga

aatcagtgactttgttaaaaagaaaaatatccctaaagatgaaacggtatatataggtggtgaccttaatgttaataaaggcactcc
agagttcaaagatatgcttaaaaacttgaatgtaaatgatgttctatatgcaggtcataatagcacatgggaccctcaatcaaattc
aattgcgaaatataattaccctaatggtaaaccagaacatttagactatatatttacagataaagatcataaacaaccaaaacaatt
agtcaatgaagttgtgactgaaaaacctaagccatgggatgtatatgcgttcccatattactacgtttacaatgatttttcagatcatt
atccaatcaaagcctatagtaaatag

>ecb
atgaaaaagaattttattgggaaatcaattttaagcatagctgctattagtttaacggtatcaacgtttgccggtgaatctcatgcaca
aactaaggttgaaaaatataacgagtatcaaacaaactttaaaaaacaagtaaataaaaaagttgtggacgcacaaaaagctgta
aacttgttcaaacgtacaagaactgttgcaacacaccgtaaagcacaaagagctgttaacttaattcatttccaacacagctatgaa
aagaaaaaattacaaagacaaatcgatctagttttaaaatataatactttaaaataa

>flipr/flipr like
atgtctaaatggattattgtgagtgataaccatactgaatcaggcgttttatatcaaatttatgaaatgcacccagatgcagatgtat
atttacatttaggagattcagaattcgcgtatgatgatacggaacttagcttatttaatagagtaaagggcaattgtgatttttatcca
gaatttgaaaatgaagcggtcgcaaaatataatgacgtgaaagcattttatactcatggacatttatatcaagtcaatcgaacaaga
gatttattagctgaaaaaggacttgaattaggttgtttgtttgcattttatggacatacacatgtggcaaaatatgagtatattaatgg
tgttcatgttattaatcctggaagtatatctcaatctagaagttcaatggaagaaacatatgctgaagttattattgatgatcaaactt
tacatggcaccatcaatttcaaaaatcgacatcacgaaacaatcagtcatactactttttaa

>efb
atgaaaaagaattttattgggaaatcaattttaagcatagctgctattagtttaacggtatcaacgtttgccggtgaatctcatgcaca
aactaaggttgaaaaatataacgagtatcaaacaaactttaaaaaacaagtaaataaaaaagttgtggacgcacaaaaagctgta
aacttgttcaaacgtacaagaactgttgcaacacaccgtaaagcacaaagagctgttaacttaattcatttccaacacagctatgaa
aagaaaaaattacaaagacaaatcgatctagttttaaaatataatactttaaaataa

>RecA
ttggataacgatcgtcaaaaagctttagatacagtaattaaaaatatggagaaatctttcggtaaaggtgccgtaatgaagttgggt
gacaatataggtcgccgagtttcaactacatcaactggttcagttacattagataatgcgctaggtgtaggtggctatcctaaaggac
gaattattgaaatttatggtcctgaaagttctggtaagacaacagtagcgcttcacgctattgctgaagtacaaagtaatggcgggg
tggcagcatttatcgatgctgaacatgctttagatccagaatatgctcaagcattaggcgtagatatcgataatttatatttatcgcaa
ccggatcatggtgaacaaggtcttgaaatcgccgaagcatttgttagaagtggtgcagttgatattgtagttgtagactcagttgctg
ctttaacacctaaagctgaaattgaaggagaaatgggagacactcacgttggtttacaagctcgtttaatgtcacaagcgttacgta
aactttcaggtgctatttctaaatcaaatacaactgctattttcatcaaccaaattcgtgaaaaagttggtgttatgttcggtaatcca
gagactacaccaggtggacgtgcattaaaattctatagttcagtaagactagaagtacgtcgtgcagaacagcttaaacaaggaca
agaaattgtaggtaatagaactaaaattaaagtcgttaaaaataaagtggcaccaccatttagagtagctgaagttgatattatgta
tggacaaggtatttctaaagagggtgaacttattgatttaggtgttgaaaacgacatcgttgataaatcaggagcatggtattcttac
aatggcgaacgaatgggtcaaggtaaggaaaatgttaaaatgtacttgaaagaaaatccacaaattaaagaagaaattgatcgta
aattgagagaaaaattaggtatatctgatggtgatgttgaagaaacagaagatgcaccaaagtcattatttgacgaagaatag

>sbi
atgaaaaataaatatatctcgaagttgctagttggggcagcaacaattacgttagctacaatgatttcaaatggggaagcaaaagc
gagtgaaaacacgcaacaaacttcaactaagcaccaaacaactcaaaacaactacgtaacagatcaacaaaaagctttttatcaa
gtattacatctaaaaggtatcacagaagaacaacgtaaccaatacatcaaaacattacgcgaacacccagaacgtgcacaagaa
gtattctctgaatcacttaaagacagcaagaacccagaccgacgtgttgcacaacaaaacgctttttacaatgttcttaaaaatgat
aacttaactgaacaagaaaaaaataattacattgcacaaattaaagaaaaccctgatagaagccaacaagtttgggtagaatcag
tacaatcttctaaagctaaagaacgtcaaaatattgaaaatgcggataaagcaattaaagatttccaagataacaaagcaccaca
cgataaatcagcagcatatgaagctaactcaaaattacctaaagatttacgtgataaaaacaaccgctttgtagaaaaagtttcaat
tgaaaaagcaatcgttcgtcatgatgagcgtgtgaaatcagcaaatgatgcaatctcaaaattaaatgaaaaagattcaattgaaa
acagacgtttagcacaacgtgaagttaacaaagcacctatggatgtaaaagagcatttacagaaacaattagacgcattagttgct
caaaaagatgctgaaaagaaagtggcgccaaaagttgaggctcctcaaattcaatcaccacaaattgaaaaacctaaagtagaat
caccaaaagttgaagtccctcaaattcaatcaccaaaagttgaggttcctcaatctaaattattaggttactaccaatcattaaaaga

ttcatttaactatggttacaagtatttaacagatacttataaaagctataaagaaaaatatgatacagcaaagtactactataatacg<br>tactataaatacaaaggtgcgattgatcaaacagtattaacagtactaggtagtggttctaaatcttacatccaaccattgaaagttg<br>atgataaaaacggctacttagctaaatcatatgcacaagtaagaaactatgtaactgagtcaatcaatactggtaaagtattatata<br>ctttctaccaaaacccaacattagtaaaaacagctattaaagctcaagaaactgcatcatcaatcaaaaatacattaagtaatttat<br>tatcattctggaaataa
>scin-B/-C<br>ttaataccaagtaatcgaatcgatttcgccgtttttaaccactggaatattttttctatgttcttctaatgggcgagtgatatcgaaggt<br>ataataaccatttttaccaccgtctttgatttttactacagctattgtattgcctttacccatctttttagctattacatataagtcttcaac<br>agttttacctttatagtgttcgtttttttcatcggcttcttttattaatttatcagcacgttcatcgtcctttttagcttggtctgctaaatttt<br>tagcgatttctaaacctttccattcataactaaagaacgccttagcatcattagtttgagttagaagacctgtagcgatgactgtagat<br>gcaataattacttttgtgatattttttttcat
>spa<br>ttatagttcgcgacgacgtccagctaataacgctgcacctaaggctaatgataatccaccaaatacagttgtaccgatgaatggattt<br>tcttcaccagtttctggtaatgcttgagctttgttagcatctgcatggtttgctggttgcttcttatcaacaacaagttcttgaccaggttt<br>gatcatgtttttatcagctaatttgttatctgcagcaattttgtcagcagtagtgccgtttgcttttgcaatgtcatttactgtatcaccag<br>gtttaacgacatgtactccgttaccatcttctttaccaggtttgttgccatcttctttgccaggcttgttgccgtcttctttaccaggttttt<br>tgttgtcttctttaccaggcttgttgccatcttctttaccaggtttttttgttgtcttctttaccaggcttgttgccgtcttctttgccaggcttg<br>ttgttgtcttctttaccaggcttgttattgtcttctttgccaggcttgttattgtcttcctcttttggtgcttgagcatcgtttagctttttagc<br>ttctgctaaaatttctttgctcactgaaggatcgtctttaaggctttggatgaagccgttacgttgttcttcagttaagttaggtaaatgt<br>aaaatttcatagaaagcattttgttgttctttgttgaatttgttgtcagcttttggtgcttgagcatcatttagctttttagcttctgctaaa<br>aggttagcgctttggcttgggtcatcttttaggctttggatgaaaccattgcgttgttcttcgtttaagttaggyawatkyaagatttca<br>tagaaagcattttgttgttctttgttgaawttgttatcmgctttcggtgcttgagattcgtttaatttttagcttcacctaaaacgttag<br>tgctttggcttgggtcgtctttaagactttgaatgaagccgttacgttgcgcttcgtttaagttaggcatgttcaagatttcatagaagg<br>cgctttgttgatctttgttgaagttattttgttgcgcatcagcttttggagcttgagagtcattaagtttttgagcttcacctaaaacgtta<br>gcactttggcttggatcatctttaaggctttggataaaaccattgcgttgatcagcatttaagttaggcatatttaagacttgataaaa<br>agcattttgttgagcttcatcgtgttgcgcagcatttgcagcaggtgttacgccaccagatataagtaatgtacctaaagttacagat<br>gcaatacctacacctagtttacgaattgaataaatgtttttctttttcaaattaatacccccctgtatgtatttgtaaagtcatcat
>ssl1<br>atgaaatttaaagcgatagcaaaagcaagtttagcattgggaatgttagcaacaggtgtaattacatcgaatgtacaatcagtaca<br>agcgaaaacagaagttaaacaacaaagtgaggctgatttaaaactttattataatggaccaagttttgaatataaaaaagtaactg<br>gatatggatttattgaaggtaaagatagatttattgattttatatacaatggacaatataataaaatatctttagttggttctgataaa<br>gataaatataatgaagaagttaacccagatatagatgtgtttgtcgttagagaaggaaacggtagacaagctgataatcattcgatt<br>ggtggcataacaaaaactaatagaggagtgtattatgactatatacacacaccaatccttgaaatcaagaaaggtaaagaagaac<br>cacaaagtagtctataccaaatttataaagaagacatctcactaaaagaacttgattttaaattaagaaagcaattaattagtcaaa<br>gtggcttgtattcaaatggtcttaaacaaggtcaaattacaattacaatgaatgatggcacaacacatacaatcgatttaagtcaaa<br>aacttgaaaaagaacgtatgggcgagtctatcgatggcagacaaatacaaaaaattctagtagaaatgaaataa
>ssl2<br>atgaaaatgaaatcaattgtaaaaataagtttgttattaggaatattagcaacaggtgtaaacactacaacggaaaaaccagttca<br>tgccgaaaagaaacctattgtaataagtgaaaatagcaaaaaattaaaagcttattatactcaacctagtattgaatataaaaatgt<br>gacaggttatatcagtttcattcaaccaagtattaaatttatgaatatcatagatggtaattctgttaataatattgctttaattggcaa<br>agataagcaacattatcatacgggtgtacatcgtaatcttaatatattttacgttaatgaggataagagatttgaaggtgcaaagtac<br>tccattggcggtatcacgagtgcaaacgataaagctgtcgacctaatagcagaagcaagagttattaaagcagatcatattggtga<br>atatgattatgactttttcccatttaaaatagataaagaagcaatgtcattgaaagagattgattttaaattaagaaaataccttattg<br>ataattatggtctttacggtgaaatgagtacagggaaaattaccgtcaaaaagaaatactacggaaagtatacatttgaattggata<br>aaaagttacaagaagaccggatgtccgatgttatcaatgtcacagatattgatagaattgaaatcaaagttagaaaagcataa
>ssl3<br>atgaaaatgagaacaattactaaaaccagtttagcactagggcttttaacaacaggcgcaattacagtaacgacgcaatcggtcaa

agcagaaaaaatacaatcaactaaagttgacaaagtaccaacgcttaaagcagagcgattagcaatgataaacataacagcagg<br>tgcaaattcagcgacaacacaagcagctaacacaagacaagaacgcacgcctaaactcgaaaaggcaccaaatactaatgagga<br>aaaaacctcagcttccaaaatagaaaaaatatcacaacctaaacaagaagagcagaaaacgcttaatatatcagcaacgccagc<br>gcctaaacaagaacaatcacaaacgacaaccgaatctacaacgcagcaaactaaaatgacaacacctccatcaacaaacacgcc<br>acaaccaatgcaatctactaaatcagacacaccacaatctccaaccataaaacaagcacaaacagatatgactcctaaatatgaa<br>gatttaagagcgtattacacgaaaccgagttttgaatttgaaaagcagtttggatttttgctcaaaccatggacgacggttaggtttat<br>gaatgttattccaaataggttcatctataaaatagctttagttggaaaagatgagaaaaaatataaagatggaccttacgataatat<br>cgatgtatttatcgttttagaagacaataaatatcaattgaaaaaatattctgtcggtggcatcacgaagactaatagtaaaaaagtt<br>aatcacaaagtagaattaagcattactaaaaaagataatcaaggtatgatttcacgcgatgtttcagaatacatgattactaagga<br>agagatttccttgaaagagcttgattttaaattgagaaaacaacttattgaaaaacataatctttacggtaacatgggttcaggaac<br>aatcgttattaaaatgaaaaacggtgggaaatatacgtttgaattacacaaaaaactgcaagagcatcgtatggcagacgtcatag<br>atggcactaatattgataacattgaagtgaatataaaataa
>ssl4<br>atgaaaataacaacaattgctaaaacaagtttagcactaggccttttaacaacaggtgtaatcacaacgacaacgcaagcagcaa<br>atgcgacaacaccgccttcaactaaagtggaaacaccgcaacaagtagcaaatgcaacaacaccatcttcaactaaagtggaagc<br>accgcaacaagcagcaaacgcgacaacaccatcttcaactaaagtagaagcaccgcaatcaaaaccaaacgcgacaacaccatc<br>ttcaactaaagtggaagcaccgcaacaagcagcaaacgcgacaacaccaccttcgtctaatgtagacacatcaccaccacaatcg<br>ccaaccacaaaacaagtaccaacagaaataaatcctaaatttaaagatttaagagcgtattatacgaaaccaagtttagaatttaa<br>aaatgagattggtattattttaaaaaaatggacgacaataagatttatgaatgttgtcccagattatttcatatataaaattgctttag<br>ttggtaaagatgataaaaaatatggtgaaggagtacataggaatgtcgatgtatttgtcgttttagaagaaaataattacaatctcg<br>aaaaatattctgtcggtggtatcacaaagagtaatagtaaaaaagttgatcacaaagcaggagtaagaattactaaggaagataa<br>taaaggtacaatctctcatgatgtttcagaattcaagattactaaagaacagatttccttgaaagaacttgattttaaattgagaaaa<br>caacttattgaaaaaaataatctgtacggtaacgttggttcaggtaaaattgttattaaaatgaaaaacggtggaaagtacacgttt<br>gaattgcacaaaaaattacaagaaaatcgcatggcagatgtcatagatggcactaatattgataacattgaagtgaatataaaata<br>a
>ssl5<br>atgaaaatggcagcaattgcgaaagcaagtttagcattaggtattttagcaacaggaacaataacgtcattgcatcaaactgtaaa<br>tgcgagtgaacatgaagcaaaatatgaaaatgtgacaaaagatatctttgacttaagagattactatagtggcgcaagtaaggaac<br>ttaaaaatgttactggttatcgttatagcaaaggtggcaagcattaccttatctttgataaacatcaaaagttcactagaatacaaat<br>ttttggtaaagatatagaaagatttaaagcacgcaaaaatccgggattagacatatttgttgttaaagaagcggaaaaccgtaatg<br>gcacagtgttttcatatggtggtgtcactaagaaaaatcaagacgcttattatgattatataaacgcaccaagatttcaaatcaaga<br>gagatgaaggtgacggtattgctacgtacggtagagtacactacatttataaagaagagatttcacttaaagaactcgactttaaat<br>tgagacagtatttaattcaaaattttgatctgtataaaaagtttcctaaagatagtaagataaaagtgataatgaaagatggcggct<br>attatacgtttgaacttaataaaaaattacaaacaaatcgcatgagtgacgtcattgacggtagaaatattgaaaaaatagaagcc<br>aacattagataa
>ssl6<br>atgaaattaaaagcgttagctaaagcaacattagtattgggattgttagctactggtgtaataacaacagaaagtcaaacagtaaa<br>agcggcagaatcaactcaaggtcaacacaattataaatcattaaaatactactatagcaagccaagtatagagttaataaatgtag<br>atggtctgtatagacaacatttaactgataaaggtgcatatgtatggaaaaatcttaaagattattatattgggctactaggtgaaga<br>tagtaagaaattcaaatcagatgtatacggggacctagatgcatttttagtcatagaagaagaacctgttaaaggaagacaatattc<br>aattggcggtataagtaagacaaatagtaaagaatttaaagaaagagaagtcgatgttaaagtaacaagaaaagcagacagaga<br>tactacatcaactaaagatagtaaatttaaaattacaaaagaagaaatctcgttaaaagagttagattttaaattaagacaaaaatt<br>gatgaaagaagagaatttatacgatgcaattaaccatagaaaaggtaaaattgtagttaaaatggaagatgataagttttatacttt<br>cgaacttacaaaaaaattacaaccgcatcgcatgggtgacacgatagatggtaccaaaatcaaagaaattaatgttgagctagaa<br>tataaataa
>ssl7<br>gtgaaattaaaaacgttagctaaagcaacattggcattaggcttattaactactggtgtgattacatcagaaggccaagcagtgcaa

gcaaaagaaaagcaagagagagtacaacatttatatgatattaaagacttacatcgatactactcatcagaaagttttgaattcagt
aatattagtggtaaggttgaaaattataacggttctaacgttgtacgctttaaccaagaaaatcaaaatcaccaattattcttatcag
gaaaagataaagataaatataaagaaggccttgaaggccagaatgtctttgtggtaaaagaattaattgatccaaacggtagact
atctactgttggtggtgtaacgaagaaaaataaccaatcttctgaaactaatacacctttatttataaaaaaagtgtatggcggaaa
tttagatgcatcaattgaatcatttttaattaataaagaagaagtttcactgaaagaacttgatttcaaaattagacaacatttagtta
aaaattatggtttatataaaggtacgactaaatacggtaagatcactttcaatttgaaagatggagaaaagcaagaaattgatttag
gtgataaattgcaattcgagcacatgggcgatgtgttgaatagtaaggatattcaaaatatagcagtgactattaatcaaatttaa

>ssl8
atgaatatgaaatttacagcgatagctaaagcgatatttgtattaggaatattaacaacaagtgtaatgataacagaaaatcaatcg
gttaatgcaaaaggaaagtatgaaaaaatgaaccgtttatatgatacaaacaagttacatcaatactattcaggacctagttatga
gttaacaaatgttagtggccaaagtcaaggttattatgactctaacgttttgctttttaaccaacaaaatcaaaagttccaagtgttttt
attgggaaaagatgaaaataaatacaaagaaaaaacacatggtttagatgtctttgcggtaccggaattagtagatttagatggaa
gaatatttagtgttagtggtgtaacaaagaaaaaatgtaaaatcaatatttgagtctctaagaacgccgaacttactagttaaaaaaa
tagacgataaagacggtttttcgtatgatgaatttttctttattcaaaaggaagaagtatcattgaaggaacttgatttcaaaataag
aaaactgttaattaaaaaatacaaattgtatgaaggggcagctgataaaggtagaattgttattaatatgaaagatgaaaataagt
atgaaattgatttaagtgataaattaggtttcgagcgtatggcagatgtcattaatagtgaacaaattaaaaacatcgaagtgaattt
gaaataa

>ssl9
atgaaatttacggcattagcaaaagcaacattagcattaggaatattaactacaggtgtgtttacaacagaaagtaaagctgttcac
gcgaaagtagaacttgatgagacacaacgcaaatattatatcaatatgctacatcaatactattctgaagaaagttttgaaccaaca
aatattagtgttaaaagcgaagattactatggctctaacgttttaaactttaaacaacgaaataaagcttttaaagtatttttacttgg
tgacgataaaaataaatataaagaaaaaacacatggccttgatgtctttgcagtacctgaattaatagatataaaaggtggcatat
atagcgttggcggtataacaaagaaaaatgtgagatcagtgtttggatttgtaagtaatccaagtctacaagttaaaaaaatcgatc
ctaaacatggcttttcgataaatgagttgttctttattcaaaaggaagaagtatcgttgaaggaactggattttaaaataagaaaaat
gttagtcgaaaaatatagattgtataaaggcgcgtcagataaaggtagaatcgttattaatatgaaagacgaaaagaaatatgtaa
ttgatttaagtgaaaaattaagttttgatcgtatgtttgatgtaatggatagtaagcaaattaaaaatattgaagtgaatttgaattaa

>ssl10
atgaaattaacagcgatagctaaagctgcattagctttaggaattttaacaacaggaactttaacaacagaagttcattcaggtcat
gcaaaacaaaatcaaaagtcagtaaataaacatgacaaggaagcattataccgatactacactggaaagactatggaaatgaaa
aatattagtgctttgaaacatggtaaaaataacttgcgttttaagtttagaggtattaagattcaagttttactgcctggaaatgataa
aagtaaatttcaacagcgtagttatgaggggttagatgtgtttttgttcaagaaaaaagagataagcacgatatattttatactgttg
gtggtgtaatacagaataataaaacatctggagttgtcagtgcaccaatattaaatatttcaaaagaaaagggtgaagatgcttttg
tgaaaggttacccttattacattaaaaaagaaaaaataacactaaaagagctggattataagttgagaaagcatctaatcgaaaa
atatggactttataaaacaatctcaaaagatggtagggtcaaaattagcttgaaagatggcagtttttataaccttgatttaagatct
aaattaaaattcaaatatatgggggaagtcatagaaagcaaacaaattaaagatattgaagttaacttaaagtaa

>ssl11
atgagtaatacacaaaagaaaaatgtgccagagttgaggttcccagggtttgaaggcgaatgggaagagaagaagttaggggac
cttactaccaaaataggtagtggaaagactcccaaaggtggaagtgaaaactatacaaacaaaggcataccatttttaaggagtc
aaaatattagaaatggtaaattaaatcttaatgacttagtttatattagtaaagatatagatgatgagatgaaaaatagtagaacgt
actatggtgatgttcttttaaatattacaggagcatcaataggtagaacagccattaattcgatagttgaaatacatgctaatttaaat
caacatgtatgtattattagattgaaaaaagagtattattataatttttttggacagtatctattatcaagaaaaggtaaaaggaaaa
ttttccttgcacaaagtggaggtagtcgagaaggactaaacttcaaagaaattgctaatttaaaaatcttcaccccaactatatttga
agagcagcaaaaaataggcgaattcatcagcaaacttgaccgacaaattgaattagaagaacaaaaacttgaattacttcagcaa
cagaaaaaaggctatatgcagaaaatcttctcgcaagaattgcgattcaaagatgaggaaggtaaagattatccagattggaaat
caaaatcaattcaagaaatatttgagaataagggtggcactgctctagaaacagaatttaattttgacggtaattataaagttataa
gtataggaagttattctataaatagcacttataatgatcaaaatataagagtcaataaaaataaaaaaactgaaaaatatattttat
caaaaggcgacttagcaatggtattaaatgataaaacaaaagatgggaaaattataggtagaagtatatttatagataaagataat

caatatatttataatcaaagaactgaaagattaataccatttgctgaaaatgataataaatttttatggttcttaatgaatacagattt<br>aattagaaataaaataaaaggtatgatgcaaggagcaacccaagtttatataaattattcatctattaaattgatatctatacaattg<br>ccacttcttgaagaacaacagaaaataagagggtttctagaagttttatctggaataactactaaacaattgcacaagatagacca<br>attaaaagagaggaaaaaggcgtttttacagaaaatgtttatttga
>ssl12<br>ttaatgctcatcaacatttggattttgaatattcaattcaaaaactttattagctacgtcaattgtaaaatcagaaccatagttgacat<br>gagctacttttaattttccatctaaataatagattgcgattgcaacatcgtaaaattcgtcaatgacaaataaactcttttcatttgtta<br>caacctcatgctctcctgagtatacaacgttaatttcccaatcattaaaaaccat
>ssl13<br>ttaatttgtcatttcttctttttcccaatcgattttatatctttctgaagaacgatctgtccatttatctttagtattggtacctttccaattt<br>gttgaagtccaatgcaattggtaatcatcacgaactcgttcgtatattacatctatatttgtttgttgtttggatgcttttctatccatagt<br>aataactgtagcgaagtctggtgaaaaccctgaagataatagagaacttgctttgttaggatcaaggaagttatctgctgctttcata<br>gaaccatttctagttttcatgaaaagttgattgccatataccgggttccaagaatctctatcatatggtccccaattttgattcaccata<br>ttgttaaatatcactttccagcctactttttatcagttgggctctctaaaattgttttgaaatcaggttgaacatatttcagtgtatgacc<br>aatcgaaacatttgcaccaataaggccgccaattttcctgtatcatcaccagtaacattaccgttgaatccataagttaaagtactc<br>atatactcttttgtatcaatcgaatttcttggatagtaatcagatatttgagctacttcattatcaggtagttgcaactgtaccttaaag<br>gctgaaggccaggctaaaccacttttgttagcaccttcttcgctataaactctatattgaccagcaatggtacctttcgttctaataact<br>agcagtttttattgtgatttttatcatcgataaaactataaaatactttttgtgcatgccattttctttatcataagtgactaaatcacc<br>tgtttttactgtagtattgcttccaatatctgtagtaccggttttaatattaatatcagaatctgcggcattagcgacaggattcattaat<br>atggaacctagcaatagtgttgttgttactgagctgactatacgtgttttcat
>ssl14<br>atgaaactattttatatcgtatttcttattattatatggctgaatatatttttaggaaatgaaatcatccatacactgactgttttaataa<br>caacattatatattgttaattcaagaaaggggattaaaaatgacagagttgaataa
>yqil<br>gcgtttaaagacgtgccagcctatgatttaggtgcgactttaatagaacatattattaaagagacgggtttgaatccaagtgagattg<br>atgaagttatcatcggtaacgtactacaagcaggacaaggacaaaatccagcacgaattgctgctatgaaaggtggcttgccaga<br>aacagtacctgcatttacagtgaataaagtatgtggttctgggttaaagtcgattcaattagcatatcaatctattgtgactggtgaa<br>aatgacatcgtgctagctggcggtatggagaatatgtctcagtcaccaatgcttgtcaacaacagtcgcttcggttttaaaatgggac<br>atcaatcaatggttgatagcatggtatatgatggtttaacagatgtatttaatcaatatcatatgggtattactgctgaaaatttagtg<br>gagcaatatggtatttcaagagaagaacaagatacatttgctgtaaactcacaacaaaaagcagtacgtgcacagcaa
>tpi<br>cacgaaacagatgaagaaattaacaaaaaagcgcacgctattttcaaacatggaatgactccaattatttgtgttggtgaaacaga<br>cgaagagcgtgaaagtggtaaagctaacgatgttgtaggtgagcaagttaagaaagctgttgcaggtttatctgaagatcaactta<br>aatcagttgtaattgcttatgagccaatctgggcaatcggaactggtaaatcatcaacatctgaagatgcaaatgaaatgtgtgcatt<br>tgtacgtcaaactattgctgacttatcaagcaaagaagtatcagaagcaactcgtattcaatatggtggtagtgttaaacctaacaa<br>cattaaagaatacatggcacaaactgatattgatggggcattagtaggtggcgca
>pta<br>gcaacacaattacaagcaacagattatgttacaccaatcgtgttaggtgatgagactaaggttcaatctttagcgcaaaaacttgat<br>cttgatatttctaatattgaattaattaatcctgcgacaagtgaattgaaagctgaattagttcaatcatttgttgaacgacgtaaagg<br>taaagcgactgaagaacaagcacaagaattattaaacaatgtgaactacttcggtacaatgcttgtttatgctggtaaagcagatg<br>gtttagttagtggtgcagcacattcaacaggagacactgtgcgtccagctttacaaatcatcaaaacgaaaccaggtgtatcaaga<br>acatcaggtatcttctttatgattaaaggtgatgtacaatacatctttggtgattgtgcaatcaatccagaacttgattcacaaggact<br>tgcagaaattgcagtagaaagtgcaaaatcagcatta
>gmk<br>cgaatatttgaagatccaagtacatcatataagtattctatttcaatgacaacacgtcaaatgcgtgaaggtgaagttgatggcgta<br>gattactttttaaaactagggatgcgtttgaagctttaatcaaagatgaccaatttatagaatatgctgaatatgtaggcaactatta<br>tggtacaccagttcaatatgttaaagatacaatggacgaaggtcatgatgtatttttagaaattgaagtagaaggtgcaaagcaagt

tagaaagaaatttccagatgcgctatttattttcttagcacctccaagtttagaacacttgagagagcgattagtaggtagaggaaca<br>gaatctgatgagaaaatacaaagtcgtattaacgaagcgcgtaaagaagttgaaatgatgaattta
>glpf<br>ggtgctgattggattgtcatcacagctggatggggattagcggttacaatgggtgtgtttgctgtcggtcaattctcaggtgcacattt<br>aaacccagcggtgtctttagctcttgcattagacggaagttttgattggtcattagttcctggttatattgttgctcaaatgttaggtgc<br>aattgtcggagcaacaattgtatggttaatgtacttgccacattggaaagcgacagaagaagctggcgcgaaattaggtgttttctct<br>acagcaccggctattaagaattactttgccaactttttaagtgagattatcggaacaatggcattaactttaggtattttatttatcggt<br>gtaaacaaaattgccgatggtttaaatcctttaattgtcggagcattaattgttgcaatcggattaagtttaggcggtgctactggtta<br>tgcaatcaacccagcacgt
>aroe<br>aattttaattctttaggattagatgatacttatgaagctttaaatattccaattgaagattttcatttaattaaagaaattatttcgaaa<br>aaagaattagatggctttaatatcacaattcctcataaagaacgtatcataccgtatttagatcatgttgatgaacaagcgattaatg<br>caggtgcagttaacactgttttgataaaagatgacaagtggatagggtataatacagatggtattggttatgttaaaggattgcaca<br>gcgtttatccagatttagaaaatgcatacattttaattttgggcgcaggtggtgcaagtaaaggtattgcttatgaattagcaaaattt<br>gtaaagcccaaattaactgttgcgaatagaacgatggctcgttttgaatcttggaatttaaatataaaccaaatttcattagcagatg<br>ctgaaaagtattta
>arcc<br>ttattaatccaacaagctaaatcgaacagtgacacaacgccggcaatgccattggatacttgtggtgcaatgtcacagggtatgata<br>ggctattggttggaaactgaaatcaatcgcattttaactgaaatgaatagtgatagaactgtaggcacaatcgttacacgtgtggaa<br>gtagataaagatgatccacgattcaataacccaaccaaaccaattggtcctttttatacgaaagaagaagttgaagaattacaaaa<br>agaacagccagactcagtctttaaagaagatgcaggacgtggttatagaaaagtagttgcgtcaccactacctcaatctatactag<br>aacaccagttaattcgaactttagcagacggtaaaaatattgtcattgcatgcggtggtggcggtattccagttataaaaaaagaaa<br>atacctatgaaggtgttgaagcg
